# Modeling Ultrafast Electron Dynamics in Strong Magnetic Fields Using Real-Time Time-Dependent Electronic Structure Methods

**DOI:** 10.1021/acs.jctc.0c01269

**Published:** 2021-03-16

**Authors:** Meilani Wibowo, Tom J. P. Irons, Andrew M. Teale

**Affiliations:** † School of Chemistry, University of Nottingham, University Park, Nottingham NG7 2RD, United Kingdom; ‡ Hylleraas Centre for Quantum Molecular Sciences, Department of Chemistry, University of Oslo, P.O. Box 1033, Blindern, N-0315 Oslo, Norway

## Abstract

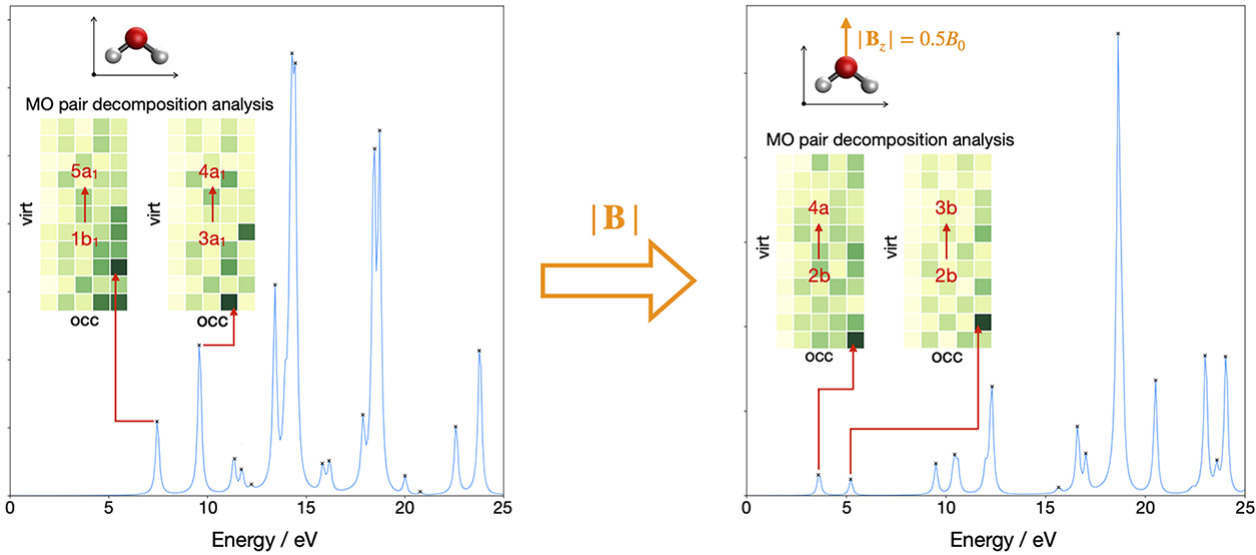

An implementation of real-time time-dependent Hartree–Fock (RT-TDHF) and current density functional theory (RT-TDCDFT) for molecules in strong uniform magnetic fields is presented. In contrast to earlier implementations, the present work enables the use of the RT-TDCDFT formalism, which explicitly includes field-dependent terms in the exchange–correlation functional. A range of current-dependent exchange–correlation functionals based on the TPSS functional are considered, including a range-separated variant, which is particularly suitable for application to excited state calculations. The performance of a wide range of propagator algorithms for real-time methods is investigated in this context. A recently proposed molecular orbital pair decomposition analysis allows for assignment of electronic transitions, providing detailed information about which molecular orbitals are involved in each excitation. The application of these methods is demonstrated for the electronic absorption spectra of N_2_ and H_2_O both in the absence and in the presence of a magnetic field. The dependence of electronic spectra on the magnetic field strength and its orientation relative to the molecule is studied. The complex evolution of the absorption spectra with magnetic field is rationalized using the molecular orbital pair decomposition analysis, which provides crucial insight in strong fields where the spectra are radically different from their zero-field counterparts.

## 1 Introduction

The development of computational methods to study light–matter interactions in the presence of external fields is essential for the understanding of fundamental photophysical processes. Light–matter interactions may generally be considered as weak-field or strong-field interactions. In the weak-field interactions, such as light harvesting in solar cells, which involves photo-excitation, electron transfer, and photodissociation processes, the external field induces only a small perturbation to the ground state electron density. As such, weak-field interactions are well described by perturbation theories such as linear response time-dependent Hartree–Fock (LR-TDHF)^1,2^ and density functional theory (LR-TDDFT),^3−5^ which yield excitation energies and oscillator strengths, and allow excitations to be described in terms of particular transitions between the molecular orbitals involved.^4^ Recently, linear response (LR) methods have been extended to treat molecular systems in the presence of strong magnetic fields at the Hartree–Fock and density functional levels^6,7^ utilizing London atomic orbitals (LAOs) so that the orbitals exhibit a physically correct response to the magnetic field, within a finite basis representation.^8^

An alternative approach to the determination of electronic absorption spectra is provided by the study of real-time (RT) electron dynamics, a review of which may be found in refs (9) and (10). Recently, implementations of RT-TDHF and RT-TDDFT have been presented for molecules in the presence of strong magnetic fields,^11^ in which the application of weak time-dependent electric fields allows the determination of electronic absorption spectra as a function of applied magnetic field. RT methods offer advantages for calculating the entire spectra of systems with a high density of states, for which LR methods would require the determination of a prohibitively large number of roots of the frequency-dependent response function. These methods can also be applied to chiroptical spectroscopies such as electronic circular dichroism (ECD) and magnetic circular dichroism (MCD) spectroscopies.^12−15^ For MCD, an implementation of RT-TDDFT methods using LAOs immediately provides a gauge-origin invariant method for the determination of MCD spectra.^15^ When a strong time-dependent electric field is applied, RT approaches can describe high-harmonic generation and multi-photon excitation processes, which involve fast non-equilibrium electron (and nuclear) dynamics^16^ that are beyond the reach of LR approaches.^17^

The utilization of DFT in RT approaches offers a favorable balance of accuracy and computational efficiency for both ground and excited states of molecules. Several implementations of RT-TDDFT, first developed by Theilhaber^18^ and pioneered by Yabana and Bertsch,^19^ utilize real-space methods, although finite basis approaches have become increasingly common.^20−22^ RT-TDDFT has been applied to a range of problems encompassing small and large molecular systems, for instance, direct integration of the Schrödinger equation for H_2_,^23^ multi-photon ionization of helium and the electronic optical response of H_2_ and N_2_,^2,24^ linear and non-linear optical response of chromophores,^13,15,22,25^ quantum dots in magnetic fields,^26^ molecular conductance,^27^ singlet–triplet transitions,^28^ core excitations,^21,29^ high-harmonic generation in photonic molecules,^16^ plasmon resonances,^30,31^ and excitations in molecular systems containing heavy elements for which the relativistic corrections have been accounted for in the four-component formalism based on the Dirac Hamiltonian.^32^ There has also been extensive work on developing schemes which go beyond the Born–Oppenheimer approximation to explicitly treat the nuclear motion, for example, *ab initio* Ehrenfest dynamics,^33^ trajectory surface hopping approaches,^34^ and wave packet propagation.^35^

The study of real-time electron dynamics in molecules with electronic structure methods however is not a simple endeavor. Calculating the time evolution of the electronic structure requires the electronic density to be propagated through time and the electronic energy to be evaluated at regular intervals. These evaluations can be computationally expensive and are particularly so in the presence of an applied magnetic field since the integration over complex LAOs is more expensive than that over real basis functions and the symmetry of the molecule is reduced by the applied field. In order to achieve sufficiently resolved spectra, the propagation must be carried out for a sufficiently long duration; typically on the order of 10^4^ time steps with periods in the range 0.05–0.20 a.u., corresponding to simulations of duration 10–50 fs. At the HF/DFT level in a finite basis, a common approach is the time propagation of the one-particle density matrix, for which a wide range of methods have been proposed.^35−37^ Each propagation algorithm provides a different compromise between accuracy, numerical stability, and computational efficiency; hence, the most appropriate algorithm may vary between applications.

Electronic absorption spectra can be obtained by applying Fourier transforms to the time-dependent dipole moments resulting from an electric field perturbation along three orthogonal axes. The positions of the peaks observed reflect the excitation energies, whilst their relative intensities describe the oscillator strengths of the associated transitions. In real-time simulations, it can be challenging to determine which orbitals are involved in each transition. However, it has recently been shown that this information may be extracted by decomposition of the contributions to the dipole signal at each time step.^32^ Furthermore, this may be combined with Padé–accelerated Fourier transformations to allow spectra to be computed with larger time steps.^38^ Nonetheless, determination of the optimal time step in a RT propagation is not a simple problem, depending on the choice of electronic structure method, propagator, and signal–processing algorithms. In the present work, this is investigated for molecular systems in the presence of strong magnetic fields.

The changes in electronic structure that can arise in the presence of strong magnetic fields is the subject of increasing interest.^39−42^ One of the most striking is the perpendicular paramagnetic bonding mechanism,^42^ which can lead to the normally repulsive triplet state of H_2_ becoming bound in a strong magnetic field oriented perpendicular to the internuclear axis. The study of excited electronic states in strong magnetic fields is of further interest since excited states have more diffuse densities, hence larger cross–sectional areas and so may be more susceptible to the influence of a magnetic field, see, for example, refs (6, 43), and (44).

The focus of the present work is the extension of RT-TDDFT methods to include the effects of strong uniform applied magnetic fields directly, utilizing the efficient integral evaluation of LAOs in our in-house program QUEST.^45,46^ Recent implementations in this context, presented using LAOs,^44^ only consider conventional density functionals. However, it has been shown that explicitly including contributions from the magnetically induced currents in the density functionals can significantly improve the description of ground state systems in strong magnetic fields.^47^ In this work, density functionals with explicit dependence on the paramagnetic current density are employed; in this implementation of real-time time-dependent current DFT (RT-TDCDFT), propagation of the density matrix allows both the charge and current density dynamics of molecular systems to be explored.

The substance of this work is organized as follows: the theoretical foundations of the RT-TDCDFT method are reviewed in Section 2. In Section 3, the range of propagator algorithms implemented is assessed for their efficiency and stability in determining the electronic absorption spectra. These are then applied to compute the electronic absorption spectra of N_2_ and H_2_O in the absence of a magnetic field and as a function of applied magnetic field, which are analyzed using the molecular orbital pair decomposition method of ref (32). Concluding remarks and directions for future work are given in Section 4.

## 2 Methodology

In this section, we present the theoretical foundations for our implementation of the RT-TDCDFT method. Atomic units are used throughout the paper, unless otherwise specified. The magnetic field strength is specified in units of *B*_0_ = ℏ*e*^–1^*a*_0_^–2^ ≈ 2.35 × 10^5^ T. We begin with a brief review of current density functional theory (CDFT) in Section 2.1 and the choice of current-dependent exchange–correlation functional. We then introduce the basic equations for RT-TDCDFT and the methods with which the time-propagation of the density matrix is achieved in Section 2.2. In Section 2.3, we discuss how the electronic absorption spectra can be computed from the RT-TDCDFT trajectories and finally we present the molecular orbital (MO) pair decomposition analysis^32,38^ for characterizing the nature of excitations.

### 2.1 Current Density Functional Theory

In the Vignale–Rasolt formulation of CDFT,^48,49^ the Kohn–Sham (KS) equations take the form
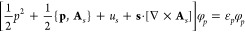
1where **p** = –i∇ is the canonical momentum operator, **s** is the spin operator, *ε_p_* are the orbital energies, and *φ_p_* are the molecular orbitals. In KS CDFT, a non-interacting system is introduced to reproduce both the charge density
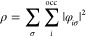
2where *i* denotes occupied orbitals and *σ* their spin, and the paramagnetic current density
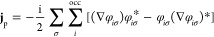
3of the physical system. The KS potentials (*u*_s_, **A**_s_) are
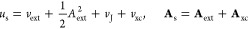
4where (*v*_ext_, **A**_ext_) are the physical external potentials, *v*_J_ is the Coulomb potential, and the exchange–correlation scalar and vector potentials are given respectively as
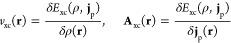
5

A central challenge for CDFT calculations is to define an exchange–correlation functional *E*_xc_(*ρ*, **j**_p_), which depends on both the charge and paramagnetic current densities. In the local density approximation (LDA), generalized gradient approximation (GGA) and hybrid-GGA levels, it is common to use the approximation *E*_xc_(*ρ*, **j**_p_) ≈ *E*_xc_(*ρ*) in response calculations. A similar approximation has been used in RT-TDDFT recently in ref (44).

In the present work, we introduce explicit current dependence at the meta-GGA level via a modification of the (gauge-dependent) kinetic energy density
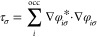
6in the manner suggested by Dobson^50^ and used by Becke^51^ and later in response calculations by Bates and Furche^52^
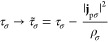
7such that the resulting exchange–correlation functional is gauge-invariant.^53^ This leads to a well-defined and properly bounded iso-orbital indicator when applied to the Tao–Perdew–Staroverov–Scuseria (TPSS) functional^54^ (see, for example, ref (55) for comparisons) and the resulting form has been called cTPSS. It was shown in ref (52) that the inclusion of the paramagnetic current density is required in response calculations using kinetic energy-dependent meta-GGA functionals, even in the absence of a magnetic field. In the present work, we use the same modification for non-perturbative calculations in the presence of external magnetic fields.

#### 2.1.1 Current-Dependent Functionals

It was shown in ref (47) that, in the presence of strong magnetic fields, the use of current-dependent meta-GGAs can avoid the significant overbinding of molecules observed at the LDA, GGA, and hybrid-GGA density functional levels. In particular, the cTPSS functional, defined by substituting the standard kinetic energy density of eq 6 with the modified form of eq 7, was shown to yield accurate interaction energies as a function of magnetic field strength. This functional incorporates the paramagnetic current density **j**_p_ at the meta-GGA level, whilst variants at the hybrid and range-separated hybrid levels were recently introduced by Irons *et al*.^56^

A simple hybrid can be constructed analogous to the TPSSh functional^57^ by admixture of 10% orbital-dependent exchange with 90% of the cTPSS exchange functional and 100% of the cTPSS correlation contribution; we denote this form cTPSSh. The construction of a range-separated analogue, denoted cTPSSrsh, follows the form introduced in ref (58). In particular, we apply the range separation to the exchange component so that the exchange energy per particle becomes
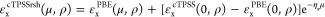
8yielding the cTPSSrsh contribution to the exchange energy
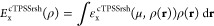
9where *ε*_x_^cTPSS^(0, *ρ*) and *ε*_x_^PBE^(0, *ρ*) are the cTPSS and PBE exchange energy densities without range separation, respectively, and *ε*_x_^PBE^(*μ*, *ρ*) is the range-separated PBE exchange energy density described in refs (59) and (60). When the range-separation parameter *μ* = 0, the standard cTPSS functional is recovered. The parameter *η_x_* is set to 15 (chosen in ref (58) to cancel the self-interaction energy of the H atom for a wide range of *μ* values). For the calculations in this work, we use *μ* = 0.4. This functional is then combined with orbital-dependent exchange integrals evaluated using the separation defined by
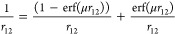
10ensuring that, for large inter-electronic separations *r*_12_, contributions from the exchange integrals, evaluated with the erf(*μr*_12_)/*r*_12_ operator, approach 100% exchange. The short-range exchange interactions are modeled by the complementary exchange component of the cTPSSrsh functional. The resulting exchange functional is combined with the standard cTPSS correlation functional.

It is well known that the nature of the exchange contribution included in density functional approximations can have a strong influence on the quality of excitation energies determined in the absence of a magnetic field. In the present work, we therefore consider results from cTPSS, cTPSSh, and cTPSSrsh. The current dependence is essential at the (hybrid) meta-GGA level to ensure gauge independence of the exchange–correlation energy and so cannot be neglected. In contrast, at the (hybrid) GGA level, the current dependence of the functional can be neglected as an approximation. We therefore also consider the standard PBE exchange–correlation functional^61^ for comparison. To help determine the influence of the orbital-dependent exchange contributions, we also consider results at the Hartree–Fock level.

### 2.2 Real-Time Time-Dependent Current Density Functional Theory (RT-TDCDFT)

The Liouville–von Neumann equation is the foundation of real-time time-dependent self-consistent field (RT-TDSCF) methods such as RT-TDHF, RT-TDDFT, and RT-TDCDFT. In an orthonormal basis, it describes the time evolution of the density matrix **P**(*t*)
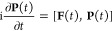
11where i is the imaginary unit, *t* is the time variable, and **F**(*t*) is the time-dependent Fock or KS matrix.

Formally, the solution to eq 11 can be written in terms of a unitary propagator
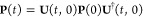
12where
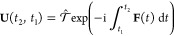
13involves time-ordered integration (denoted by ). Since **U** is unitary, properties present in **P**(0), such as idempotency and trace (particle number), are preserved for times *t* > 0.

In practice, eq 11 is solved by discretizing time into small time steps Δ*t* such that the propagator at time step *N* is described by **U***_N_* = **U**(*t_N_* + Δ*t*, *t_N_*) and propagates **P**(*t*) to **P**(*t* + Δ*t*). The time ordering of the integration in eq 11 can be neglected for sufficiently small time steps, with the exact time ordered propagator obtained as Δ*t* → 0. In a finite basis set of LAOs, as used in this work, the required matrix exponentials can be evaluated by diagonalization and the dominant cost is then the formation of the Fock/KS matrices **F**(*t*). In the present work, we utilize the implementation of CDFT in our in-house program QUEST^46^ to construct the required **F**(*t*) for Hartree–Fock and CDFT calculations. In particular, both scalar and vector potential contributions arising from current-dependent density functionals are included in the propagation. Since the construction of **F**(*t*) already uses complex algebra for HF/(C)DFT in magnetic fields, the implementation of RT-TDCDFT requires only the construction and application of approximations to **U***_N_* using existing routines.

Many algorithms have been put forward for the construction of approximate **U***_N_* and their application to propagate **P**(*t*) to **P**(*t* + Δ*t*). In this work, we consider the Magnus 2,^62^ Magnus 4,^62^ modified midpoint unitary transformation (MMUT),^24^ and exponential density predictor/corrector (EPPC-*N*)^63^ propagators.

#### 2.2.1 Magnus Expansion-Based Propagators

The Magnus expansion-based propagators are constructed by considering the Magnus expansion^64^ of the propagator in eq 13
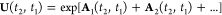
14in which the first two terms are given as
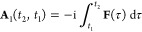
15
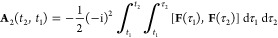
16

The propagator in eq 13 can be approximated for small time intervals by truncating eq 14 and using simple numerical quadrature rules, such as the midpoint or trapezoidal rules. The simplest Magnus 2 propagator can thus be approximated as
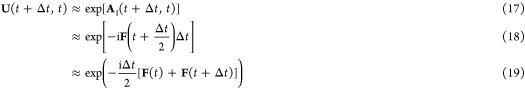
where eq 18 uses the midpoint rule and eq 19 uses the trapezoidal rule.

In our implementation, we use eq 19, where, at a given time step, **F**(*t*) is evaluated from density matrix **P**(*t*) and then an approximate **P̃**(*t* + Δ*t*) is evaluated using the Euler approximation **P̃**(*t* + Δ*t*) = exp[–iΔ*t***F**(*t*)]**P**(*t*)exp[–iΔ*t***F**(*t*)]^†^; this density matrix is then used to evaluate **F**(*t* + Δ*t*) and eq 19, from which the resulting **U** is then used for the final propagation of the density matrix to generate **P**(*t* + Δ*t*) for the next time step. This requires two Fock/KS matrix constructions and two matrix exponential evaluations per time step. The errors in this approach are  and, in our experience, the integration is stable for modest time steps in the range 0.05–0.20 a.u. It has been suggested that the stability of the approach can be further improved by predictor–corrector algorithms,^22,27,32^ but we do not explore this in the present work.

The Magnus 4 propagator can be derived in a similar manner by including more terms in eq 14. In practice, higher order Magnus schemes are rarely used because the expressions involve increasingly complicated integrals over nested commutators. We have implemented the Magnus 4 scheme in our program to serve as a benchmark, but this scheme requires six Fock/KS matrix constructions and two matrix exponential evaluations per time step, making it too expensive for general use.

#### 2.2.2 Modified Midpoint Unitary Transformation (MMUT) Propagation

One of the most efficient approaches to perform real-time density matrix propagation is the modified midpoint unitary transformation (MMUT) approach of Li *et al.*(24) In this approach, the propagator is approximated by
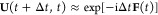
20which is used to propagate the density matrix  to ,
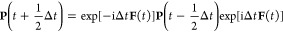
21from which the density matrix at the next time step is determined by
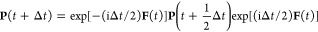
22

The MMUT approach requires only one Fock/KS matrix construction per time step, making it the most efficient of the methods considered in this work. Errors are formally of ; however, the leapfrog nature of the integration means that it must be started using a non-leapfrog method. In the present work, the Magnus 2 propagator is used to start the MMUT procedure. In practice, the MMUT procedure can suffer from energy drift unless it is restarted periodically;^44^ here, the Magnus 2 propagator is also used for this purpose. In the present work, the MMUT propagator is restarted every 50 steps.

#### 2.2.3 Exponential Density Predictor/Corrector (EPPC) Propagation

Recently, Zhu and Herbert proposed a family of predictor/corrector type propagation algorithms, with the exponential density predictor/corrector (EPPC) methods proposed for general use.^63^ We have implemented the EPPC-1, EPPC-2, and EPPC-3 methods in the present work. In these approaches, **F**(*t*) and **P**(*t*) are used to form the predictor density
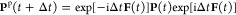
23with which **F**^p^(*t* + Δ*t*) is constructed to form the propagator
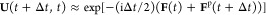
24from which the corrector density is obtained as
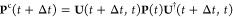
25

The predictor and corrector densities are then compared via the Frobenius norm ∥**P**^p^(*t* + Δ*t*) – **P**^c^(*t* + Δ*t*)∥ and if this value is above a given threshold then **P**^p^(*t* + Δ*t*)←**P**^c^(*t* + Δ*t*); **F**^p^(*t* + Δ*t*) is updated accordingly and the process is iterated until the predictor and corrector densities agree to within a required threshold. The three variants of the EPPC method then differ only by how the density matrix for the next time step is constructed. EPPC-1 uses **P**(*t* + Δ*t*) = **P**^c^(*t* + Δ*t*), where **P**^c^(*t* + Δ*t*) is the latest corrector density matrix. EPPC-2 uses **P**(*t* + Δ*t*) = [**P**^p^(*t* + Δ*t*) + **P**^c^(*t* + Δ*t*)]/2, which corresponds to the average of the last two evaluations of the predictor and corrector density matrices. EPPC-3 uses the average of the first evaluation of the predictor density matrix at a given time step and the final value of the corrector density matrix, **P**(*t* + Δ*t*) = [**P**^p[0]^(*t* + Δ*t*) + **P**^c^(*t* + Δ*t*)]/2.

The iterative nature of the predictor/corrector algorithms means that a number of Fock/KS matrix constructions are required at each time step; the efficiency of the approach can be measured by an average of the number of such constructions per time step over the course of a simulation. Overall, the efficiency of the EPPC approaches will depend on the trade-off between the enhanced stability of the approach for larger time steps and the number of Fock/KS matrix constructions per time step.

### 2.3 Computing Electronic Absorption Spectra from RT-TDSCF Simulations

To compute the electronic absorption spectra, first we need to perform three independent RT-TDSCF calculations for each Cartesian direction *α* ∈ {*x*, *y*, *z*} of an external electric field pulse. In the present work, we have used an electric field
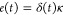
26where *κ* is the electric field strength and the Dirac delta function *δ*(*t*) imposes that the perturbation of the electric field is applied only at *t* = 0. For an electric field applied along Cartesian direction *α*, the three Cartesian components *β* of the time-dependent electric dipole moment are computed as
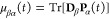
27where **D***_β_* is the matrix representation of the *β* component of electric dipole operator and **P***_α_*(*t*) is the time-dependent density matrix in the AO basis for an electric field applied along *α*. The diagonal elements of the field-dependent complex polarizability tensor, *α_αα_*(*ω*), are then computed from the Fourier transformation of the time-dependent dipole moment as
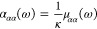
28

Finally, the electronic absorption spectrum (the dipole strength function), *S*(*ω*), is computed from the polarizability tensor as
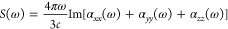
29

### 2.4 Molecular Orbital (MO) Pair Decomposition Analysis

The computed RT-TDSCF spectra as described in Section 2.3 do not provide any information about the nature of the excitations, i.e., which MOs are involved in a particular excitation. However, this information is contained in the time-dependent density matrix. It can be extracted using the MO pair decomposition analysis.^32,38^ In this analysis, the time-dependent density and dipole matrices in the AO basis are transformed to the ground state MO basis
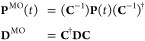
30where **C** is the vector of MO coefficients at *t* = 0.

To study the electronic transitions and induced dipole moments, the time-dependent dipole moment in eq 27 can be rewritten in the MO basis (suppressing the notation for the Cartesian components for clarity) as
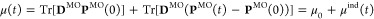
31where *μ*_0_ is the static dipole moment of the system and *μ*^ind^(*t*) is the time-dependent induced dipole moment. The initial density matrix **P**^MO^(0) at *t* = 0 is diagonal, being equal to the identity matrix in the occupied–occupied block and zero for all other blocks. The total time-dependent induced dipole moment can be decomposed into contributions from the individual occupied (*i*)–virtual (*a*) MO pairs.^32,38^ For each pair *ia*, the contribution is
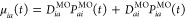
32

As a result, eq 31 can be rewritten as
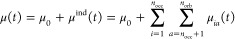
33where *n*_occ_ is the total number of occupied orbitals. In practice, the Fourier transformations of each *μ_ia_*(*t*) yield MO pair contributions to each excitation in the molecular system, giving qualitative information similar to the excitation amplitudes obtained from LR-TDSCF calculations.

## 3 Results and Discussion

The RT-TDSCF methods described in Section 2 have been implemented in our in-house program QUEST.^46^ Current-dependent cTPSS, cTPSSh, and cTPSSrsh functionals were used in RT-TDCDFT calculations. For comparison, RT-TDDFT calculations with the standard PBE exchange–correlation functional were also carried out, along with RT-TDHF calculations.

The optimized geometry of N_2_ and H_2_O, obtained at the B3LYP/aug-cc-pVTZ and PBE0/aug-cc-pVTZ levels of theory, respectively, were used. The RT-TDSCF calculations were carried out using a *δ*-function-type pulse at *t* = 0 with electric field strength *κ* = 0.0001 a.u. The 6-31G basis set was used for the investigation of the efficiency and stability of the propagators, whilst the larger 6-311++G** basis set, which includes polarization and diffuse functions, was used to compute the electronic absorption spectra of N_2_ and H_2_O both in the absence and in the presence of a magnetic field. The propagations were carried out for a total simulation time of 48 fs, leading to an energy resolution of ±0.09 eV in the calculated electronic absorption spectra. The computed spectra were artificially broadened with an exponential function, giving well-defined Lorentzian lineshapes with a full-width half-maximum (FWHM) value of 0.2 eV.

In this section, we begin by discussing the selection of an appropriate time step and propagator for calculations in the presence of a magnetic field. The influence of the external magnetic field on the required time step and the stability of the propagation is investigated. Once appropriate parameters are established, the spectra of the N_2_ and H_2_O molecules are calculated as a function of magnetic field. The influence of the choice of (C)DFT functional on the absorption spectra in magnetic fields is investigated. The utility of the MO pair decomposition analysis is demonstrated in this context, allowing for the assignment of transitions at each field strength and enabling us to follow how these transitions evolve as a function of magnetic field.

### 3.1 Efficiency and Stability of the Propagators

To investigate the efficiency and stability of the propagators introduced in Section 2.2, we performed RT-TDSCF simulations using three propagator algorithms based on the second-order Magnus expansion (Magnus 2), modified midpoint unitary transformation (MMUT), and exponential density predictor/corrector (EPPC-*N*, particularly the EPPC-1 and EPPC-3 methods since the EPPC-2 method shows a very similar performance to the EPPC-1 method^63^) in conjunction with various integration time steps ranging from Δ*t* = 0.05 a.u. (0.0012 fs) to Δ*t* = 1.0 a.u. (0.024 fs). In each case, the total simulation time is maintained at 48 fs.

For comparative and illustrative purposes, we only present the results and analysis for the N_2_ molecule, and similar trends are observed for other systems in our experience. Whilst the MMUT and Magnus 2 methods require only one and two Fock/KS matrix constructions per time step, respectively, the EPPC-1 and EPPC-3 methods require a minimum of two Fock/KS matrix constructions per time step regardless of the choice of level of theory (see Table 1). Moreover, the average number of Fock/KS matrix constructions per time step computed using EPPC-1 and EPPC-3 increases as the integration time step becomes larger. The inclusion of an external magnetic field in the RT-TDSCF simulations does not significantly change the average number of Fock/KS matrix constructions computed using EPPC-1 and EPPC-3, indicating that the application of a magnetic field does not complicate the real-time propagation (see Table S1 in the Supporting Information).

**Table 1:** Average Number of Fock/Kohn–Sham Matrix Constructions per Time Step for N_2_, Computed Using the EPPC-1 and EPPC-3 Propagator Algorithms with Various Methods and Time Steps in the Absence of a Magnetic Field^a^

	HF		cTPSS		cTPSSh		cTPSSrsh	
Δ*t* (a.u.)	EPPC-1	EPPC-3	EPPC-1	EPPC-3	EPPC-1	EPPC-3	EPPC-1	EPPC-3
0.05	2.00	2.00	2.00	2.00	2.00	2.00	2.00	2.00
0.10	2.00	2.00	2.00	3.22	2.00	2.43	2.00	2.00
0.20	3.41	2.00	2.00	4.20	3.86	3.86	2.00	2.00
0.25	3.27	2.00	2.00	4.51	2.00	4.33	2.76	2.00
0.50	3.00	2.01	2.33	5.44	2.55	5.62	3.10	2.01
1.00	6.28	2.06	5.74	7.24	5.82	7.13	7.22	2.11

a The total simulation time is 48 fs in each case.

We computed the maximum absolute values of the fluctuation in the total energy for RT-TDSCF simulations of N_2_ to further investigate the stability of each propagator. The results are presented in Table 2. The Magnus 2 and EPPC-1 algorithms are relatively stable for all integration time steps and levels of theory used in this work. However, the computed absorption spectra obtained using Δ*t* ≥ 0.5 a.u. start to deviate from those obtained using smaller time steps (see Figure S1 in the Supporting Information).

**Table 2:** Order of Magnitude for Maximum Absolute Deviation of the Total Energy from its Initial Value (in a.u.) for RT-TDSCF Simulations of N_2_, Computed Using Four Different Propagator Algorithms for Various Time Steps and Electronic Structure Methods^a^

	HF				cTPSS			
Δ*t* (a.u.)	Magnus 2	MMUT	EPPC-1	EPPC-3	Magnus 2	MMUT	EPPC-1	EPPC-3
0.05	10^–10^	10^–9^	10^–10^	10^–10^	10^–9^	10^–9^	10^–10^	10^–1^
0.10	10^–9^	10^–9^	10^–10^	10^–10^	10^–9^	10^–9^	10^–10^	10^1^
0.20	10^–9^	10^1^	10^–7^	10^–10^	10^–9^	10^0^	10^–10^	10^1^
0.25	10^–9^	10^1^	10^–7^	10^–9^	10^–9^	10^–8^	10^–10^	10^1^
0.50	10^–9^	10^1^	10^–9^	10^–9^	10^–8^	10^–5^	10^–9^	10^1^
1.00	10^–8^	10^1^	10^–8^	10^–8^	10^–9^	10^–7^	10^–8^	10^1^

	cTPSSh				cTPSSrsh			
Δ*t* (a.u.)	Magnus 2	MMUT	EPPC-1	EPPC-3	Magnus 2	MMUT	EPPC-1	EPPC-3
0.05	10^–10^	10^–9^	10^–10^	10^–8^	10^–9^	10^–9^	10^–11^	10^–10^
0.10	10^–10^	10^–9^	10^–10^	10^–4^	10^–9^	10^–9^	10^–10^	10^–10^
0.20	10^–10^	10^–9^	10^–10^	10^0^	10^–10^	10^–8^	10^–10^	10^–10^
0.25	10^–9^	10^–8^	10^–10^	10^0^	10^–9^	10^–4^	10^–8^	10^–9^
0.50	10^–9^	10^–8^	10^–9^	10^0^	10^–9^	10^–6^	10^–8^	10^–9^
1.00	10^–9^	10^–7^	10^–8^	10^0^	10^–8^	10^0^	10^–8^	10^–8^

a The total simulation time is 48 fs in each case. Values of 10^–7^ a.u. or smaller are considered acceptable.

Despite being the most efficient algorithm presented in this work, MMUT is no longer stable for Δ*t* ≥ 0.2 a.u. since the maximum fluctuation in the total energy increases significantly (see Table 2); in accordance with refs (24) and (65), which suggest that a typical time step of ∼0.05–0.1 a.u. should be used in MMUT simulations. Interestingly, EPPC-3 shows strong dependence on the nature of the electronic structure method employed. This propagator performs well for the HF and cTPSSrsh levels of theory, which contain large amounts of orbital-dependent exchange for which the maximum absolute deviation of the total energy from its initial value oscillates between 10^–10^ and 10^–8^ a.u. (see Table 2). In contrast, it is relatively unstable for the cTPSS and cTPSSh functionals, even using a small time step.

Overall, the Magnus 2 and EPPC-1 methods display a good trade-off between stability and efficiency. Both are relatively stable for all integration time steps and levels of theory used in this work. Whilst Magnus 2 requires only two Fock/KS matrix constructions per time step regardless of the step size, EPPC-1 may need more than two Fock/KS matrix constructions per time step, particularly for larger time steps. However, using a modest time step of Δ*t* = 0.1 a.u., the EPPC-1 method requires an average of two Fock/KS matrix evaluations per time step, making it comparable with the Magnus 2 method. In the remainder of this work, we therefore opt to use the Magnus 2 approach with this time step to ensure sufficient spectral resolution, though we note that, if less of the spectral range is required, EPPC-1 with a larger time step could be a useful alternative, in line with the observations in ref (63).

### 3.2 Electronic Absorption Spectra of N_2_ and H_2_O at Zero Magnetic Field

We commence by considering the electronic absorption spectra of N_2_ and H_2_O in the absence of a magnetic field. The electronic configuration of the N_2_ ground state obtained from all the DFT calculations used in this work, (1*σ_g_*)^2^(1*σ_u_*)^2^(2*σ_g_*)^2^(2*σ_u_*)^2^(1*π_u_*)^4^(3*σ_g_*)^2^, differs from that obtained from the HF calculation by switching the order of 1*π_u_* and 3*σ_g_* orbitals. However, the virtual orbitals obtained from both HF and DFT calculations have the same order with the degenerate 1*π_g_* orbitals being the lowest in energy. The orbital ordering obtained with DFT corresponds to the experimentally observed order of the outer valence ionization potentials.^66^ Previous theoretical studies have been performed using LR-TDDFT, typically reporting only excited states with energies below 15 eV.^67−69^

In the LR-TDDFT formulation, the excitation energies and oscillator strengths are obtained as the poles and residues of the frequency-dependent polarizability. Hence, all excited states can be determined using this method, regardless the transition probability.^4^ In contrast, the RT approach is based on a perturbation of the dipole moment and can only determine excited states with non-vanishing transition dipole moment. As a consequence, excited states with low or zero transition dipole moment will not be observed in our calculations.

**Figure 1:**
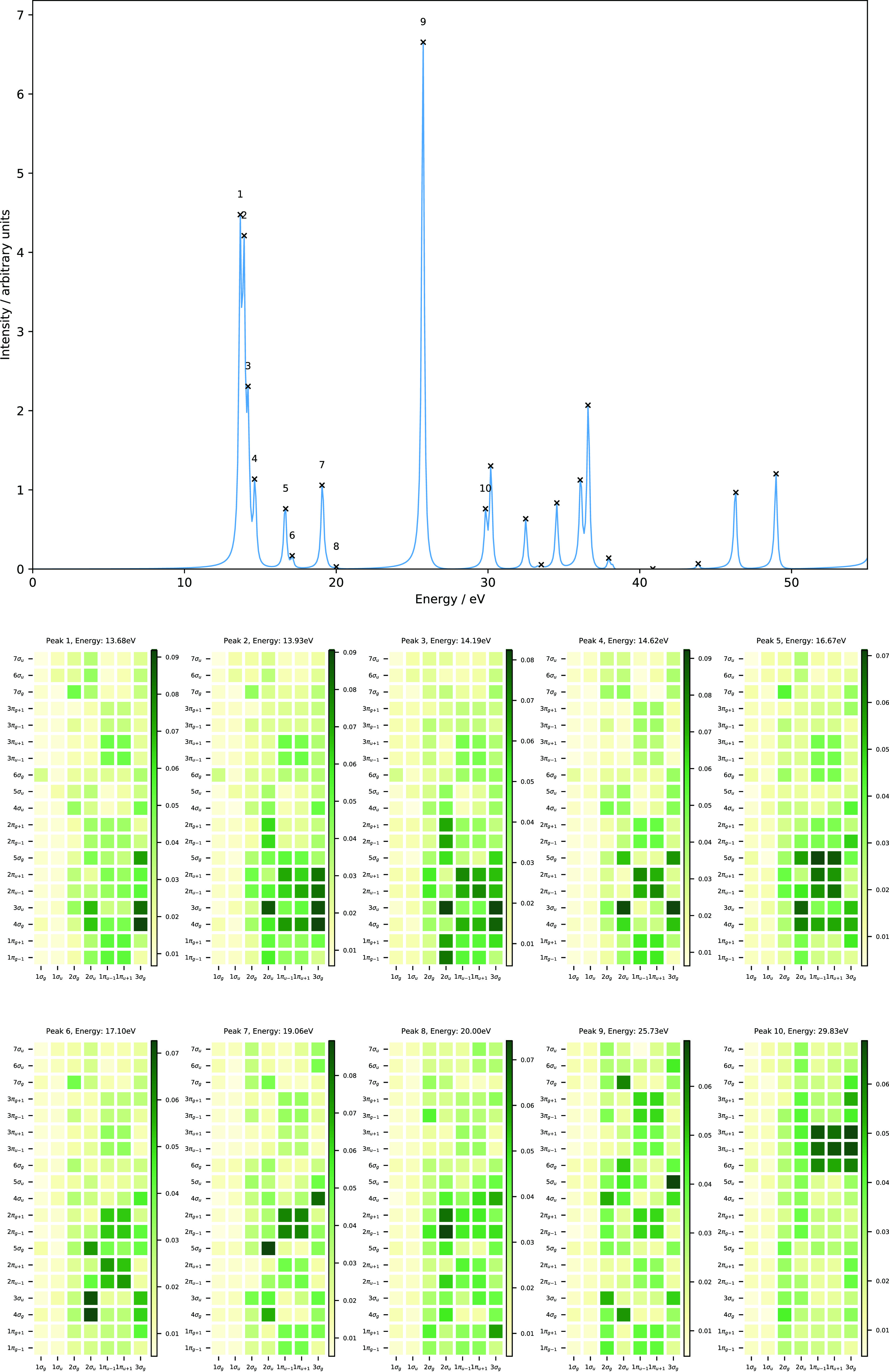
Computed absorption spectrum of N_2_ (top) and the corresponding MO pair contributions (bottom), obtained from the RT-TDDFT simulations employing the cTPSSrsh functional and 6-311++G** basis set at zero magnetic field.

The computed absorption spectra of N_2_ with excitation energies below 55 eV obtained from our RT-TDCDFT calculations are presented in Figure 1, and the corresponding data for the 10 lowest singlet vertical excitation energies are collected in Table 3. Additionally, we have also computed the excitation energies (and oscillator strengths) of these states using LR-TDHF in order to compare with our RT-TDHF results. The computed excitation energies obtained using RT-TDHF are in good agreement with those obtained from the LR-TDHF calculations (see Table 3), in line with a spectral resolution of ±0.09 eV.

**Table 3:** The 10 Lowest Singlet Vertical Excitation Energies (Δ*E*, in eV) of N_2_ and H_2_O, Computed Using the RT-TDHF and RT-TDDFT Methods in the Absence of an External Magnetic Field^c^

		Δ*E*								
molecule	peak	LR-TDHF	RT-TDHF	PBE	cTPSS	cTPSSh	cTPSSrsh	EOM-CCSD	exp.	assignment^a^
N_2_	1	14.30 (0.89)	14.28	12.31	12.57	12.99	13.68	13.69		3*σ_g_*→4*σ_g_*
	2	15.21 (0.04)	15.22	13.25	12.82	13.16	13.93	13.72		3*σ_g_*→4*σ_g_*
	3	15.96 (0.22)	15.99	13.51	13.42	13.59	14.19	13.97		2*σ_u_*→3*σ_u_*
	4	16.94 (0.12)	16.93	13.85	13.93	14.10	14.62	14.61		3*σ_g_*→3*σ_u_*
	5	19.88 (0.09)	19.92	15.47	15.56	15.81	16.67			1*π_u_*→5*σ_g_*
	6	21.47 (0.08)	21.46	17.10	17.61	18.04	17.10			2*σ_u_*→4*σ_g_*
	7	27.19 (1.17)	27.18	18.46	18.55	18.72	19.06			2*σ_u_*→5*σ_g_*
	8	30.39 (0.04)	30.43	24.45	24.53	24.87	20.00^b^			2*σ_u_*→2*π_g_*
	9	31.86 (0.18)	31.89	28.64	29.06	29.32	25.73			3*σ_g_*→5*σ_u_*
	10	34.62 (0.02)	34.62	29.32	29.58	29.75	29.83			3*σ_g_*→3*π_u_*
H_2_O	1	8.57 (0.04)	8.55	6.41	6.58	6.84	7.44	7.38	7.4	1*b*_1_→5*a*_1_
	2	10.90 (0.11)	10.94	8.55	8.80	9.06	9.57	9.77	9.7	3*a*_1_→4*a*_1_
	3	12.56 (0.04)	12.56	10.00	10.17	10.51	11.37	11.52	11.46	3*a*_1_→2*b*_2_
	4	14.26 (0.17)	14.28	10.68	10.86	11.11	11.71	11.84	11.53	1*b*_1_→5*a*_1_
	5	15.34 (0.02)	14.96	11.11	11.71	11.97	12.22			1*b*_1_→3*b*_2_
	6	15.74 (0.23)	15.73	11.54	12.74	12.99	13.42			1*b*_2_→4*a*_1_
	7	17.10 (0.01)	17.10	12.48	13.34	13.67	14.28			1*b*_2_→2*b*_2_
	8	17.32 (0.01)	17.35	13.16	13.68	13.93	14.45			1*b*_2_→2*b*_2_
	9	17.67 (0.02)	17.69	13.42	14.70	15.04	15.81			1*b*_2_→2*b*_2_
	10	18.72 (0.03)	18.72	14.53	15.47	15.73	16.16			3*a*_1_→4*b*_2_

a This assignment is for the cTPSSrsh functional, considered throughout the rest of this work. It reflects the most dominant MO pair contribution; for minor contributions see the text and Figures 1 and 2.

b Note that this peak does not have sufficient oscillator strength to appear in the peak picking for HF and the other DFT functionals considered here.

c The computed excitation energies (and oscillator strengths) obtained using LR-TDHF are also presented. For comparison, the first four excitation energies computed in the absence of a magnetic field with EOM-CCSD using the Psi4 code^70^ are given for both molecules.

The HF excitation energies tend to be significantly higher than the DFT excitation energies. In line with this, the excitation energies for the DFT functionals are in the order cTPSS < cTPSSh < cTPSSrsh, reflecting the increasing contribution from orbital-dependent exchange. The excitation energies computed using the standard PBE exchange–correlation functional are qualitatively similar to those obtained from the cTPSS functional. The resulting HF spectra have a qualitatively different appearance to those from DFT, with peaks 1 and 2 being well separated in HF, and similarly for peaks 3 and 4 (see Table 3).

To understand the nature of excitations for each excited state in N_2_, we also performed the MO pair decomposition analysis and the results are presented in Figure 1. From the MO pair decomposition analysis, the four lowest singlet excited states of N_2_, which lie close in energy, are dominated by a transition from the 3*σ_g_* orbital to the 3*σ_u_* orbital. For each state, there are some additional transitions that result in a mixed character, as shown in Figure 1. For instance, peak 1 also consists of a transition with a 3*σ_g_*→4*σ_g_* character, whilst peak 2 consists of transitions with 3*σ_g_*→4*σ_g_*,2*π_u_*, 2*σ_u_*→3*σ_u_*, and 1*π_u_*→4*σ_g_* characters. The mixing of excitations in peak 3 resembles that of peak 2 with an additional contribution from the 2*σ_u_*→1*π_g_* transition, whilst mixing is less pronounced for peak 4. Peak 5, on the other hand, is dominated by a transition from the degenerate 1*π_u_* orbitals to the 5*σ_g_* orbital and minor contributions from transitions with 2*σ_u_*→4*σ_g_*,3*σ_u_* and 1*π_u_*→2*π_u_* characters.

Even in this relatively simple case, the utility of the MO pair decomposition analysis is clear, allowing assignment of the peaks and showing that although the occupied orbital energies re-order between HF and DFT, the excitations occur in the same order and involve similar orbitals. The peaks show a strongly mixed character for N_2_; this is consistent with analysis from linear response calculations, validating the MO pair decomposition approach and reflecting the high spectral density for this molecule.^67−69^

The 10 lowest singlet excited states of H_2_O with excitation energies below 35 eV computed using RT-TDSCF simulations are collected in Table 3. We have also computed the excitation energies (and oscillator strengths) of these states using LR-TDHF in order to compare with our RT-TDHF results. The computed excitation energies obtained using RT-TDHF are in good agreement with those obtained from the LR-TDHF calculations (see Table 3). These energies are significantly higher than the DFT excitation energies. The PBE and cTPSS functionals, which contain no contribution from orbital-dependent exchange, result in underestimation of the excitation energies. The inclusion of 10% orbital-dependent exchange in the hybrid cTPSSh functional slightly improves the excitation energies. Increasing the contribution from the orbital-dependent exchange in the range-separated cTPSSrsh functional yields excitation energies that are in very good agreement with the available reported experimental data.^71,72^

Overall, the RT-TDHF and LR-TDHF values are in excellent agreement confirming the accuracy of the selected time step and propagator. The RT-TDDFT values are generally closer to experiment than the HF values but still deviate from experiment by about 10%. The exceptions being those obtained using the cTPSSrsh functional, which are in close agreement with the available experimental data as well as with the computed excitation energies obtained using EOM-CCSD. We will therefore use this functional for the remainder of this work.

The ground state of H_2_O with *C*_2*v*_ symmetry has the electronic configuration (1*a*_1_)^2^ (2*a*_1_)^2^ (1*b*_2_)^2^ (3*a*_1_)^2^ (1*b*_1_)^2^, where the 1*a*_1_ and 2*a*_1_ orbitals correspond to the 1s and 2s orbitals of the oxygen atom. The valence orbitals comprise the occupied 1*b*_2_, 3*a*_1_, and 1*b*_1_ orbitals and the two lowest unoccupied anti-bonding orbitals of *a*_1_ and *b*_2_ symmetries (4*a*_1_ and 2*b*_2_ orbitals). The 1*b*_2_ and 3*a*_1_ orbitals are the O–H bonding and non-bonding orbitals, whilst the highest occupied molecular orbital (HOMO), 1*b*_1_ orbital, is a non-bonding orbital related to the 2p*_x_* orbital of the oxygen atom (the lone pair of the oxygen atom).

The computed absorption spectrum of H_2_O and the corresponding MO pair contributions to each peak are presented in Figure 2. Peak 1, which appears at 7.44 eV, is dominated by the HOMO → LUMO+2 transition (1*b*_1_→5*a*_1_), corresponding to the one-electron promotion from the lone pair of the oxygen atom to the O–H anti-bonding orbital, with a minor contribution from the 1*b*_1_→4*a*_1_ transition. Peak 2 has a dominant contribution from the HOMO-1 → LUMO transition (3*a*_1_→4*a*_1_), which corresponds to the one-electron excitation from the O–H non-bonding orbital to the O–H anti-bonding orbital, and a minor contribution from HOMO → LUMO+4 (1*b*_1_→3*b*_2_). Peak 3 consists of excitation from HOMO-1 → LUMO+1 (3*a*_1_→2*b*_2_), describing one-electron promotion from the O–H bonding orbital to the O–H anti-bonding orbital, and a minor contribution from the 3*a*_1_→4*b*_2_ transition. Peak 4 shows a mixed character, describing transitions from HOMO → LUMO+2, LUMO+3, LUMO+4, and LUMO+5 (1*b*_1_→5*a*_1_,2*b*_1_,3*b*_2_, and 6*a*_1_) and from HOMO-1 → LUMO (3*a*_1_→4*a*_1_). The nature of excitations for peak 5 is similar to that of peak 4 with less mixing, for which the 1*b*_1_→3*b*_2_ and 3*a*_1_→4*a*_1_ transitions are the dominant ones. Again, the MO pair decomposition analysis is very useful to determine the nature of excitations, characterizing the MO pairs contributing to each transition. In contrast to the N_2_ molecule, most of the peaks are more strongly dominated by a single MO pair transition, resulting in a simpler spectrum in the energy range considered and again these are consistent with assignments from LR-TDDFT calculations.

**Figure 2:**
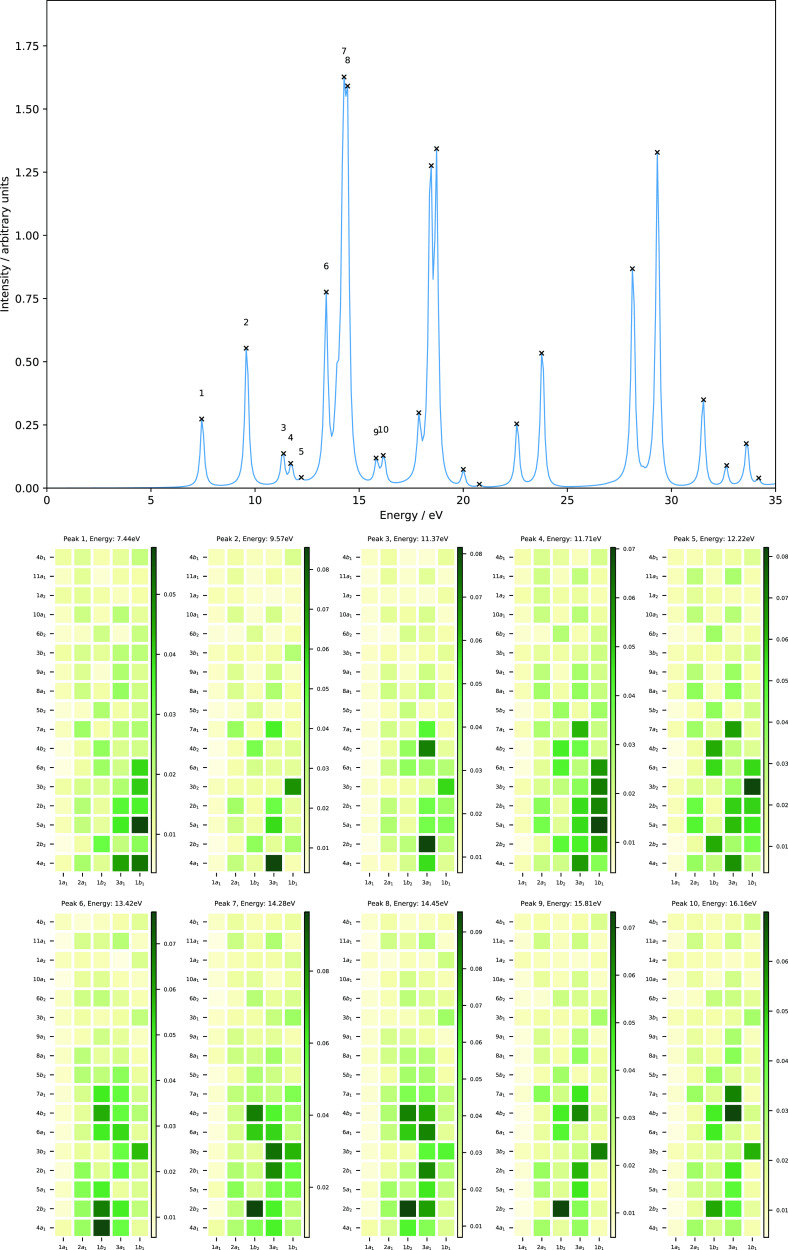
Computed absorption spectrum of H_2_O (top) and the corresponding MO pair contributions (bottom), obtained from the RT-TDDFT simulations employing the cTPSSrsh functional and 6-311++G** basis set at zero magnetic field.

### 3.3 Effects of Magnetic Field on the Computed Electronic Absorption Spectra of N_2_ and H_2_O

External magnetic fields can dramatically change the electronic structure of atoms and molecules. In particular, for excitation energies, the application of a magnetic field can break the spatial symmetry of molecular orbitals, making some symmetry forbidden transitions allowed. In Section 3.2, the MO pair decomposition was used in conjunction with RT-TDSCF calculations on N_2_ and H_2_O at zero magnetic field to validate our implementation against LR-TDHF calculations. The utility of this approach for assigning the character of the transitions was apparent in cases where the transition energies were similar. In the presence of a magnetic field, degeneracies are lifted and orbital re-orderings are common and so significant changes in the electronic absorption spectra are expected. In this section, we investigate the effects of the magnetic field on the computed spectra of N_2_ and H_2_O, demonstrating further the utility of the MO pair decomposition analysis.

In general, when an external magnetic field is applied to a molecule, the energy may either decrease paramagnetically or increase diamagnetically. Paramagnetism is often (though not always^73^) associated with open-shell molecules, whilst diamagnetism is associated with closed-shells. The electronic Hamiltonian in the presence of a uniform magnetic field **B** is
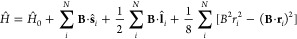
34

The first term is the unperturbed electronic Hamiltonian. The linear Zeeman terms are associated with the spin (**ŝ***_i_*) and orbital angular momentum (**l̂***_i_* = –I**r***_i_* × **∇***_i_*) operators, describing the interaction of the electron *i* with the magnetic field **B**. These terms may split the energy levels of atoms and molecules and raise or lower the total energy relative to the energy at zero field. Together, these spin and orbital terms are often called the paramagnetic Zeeman terms. The remaining term always raises the energy relative to zero field. Since it is quadratic in **B**, then, at sufficiently large fields, it will always dominate, meaning that eventually the energy of a molecular system will increase diamagnetically. This term is associated with the interaction of the magnetic moment induced by Larmor precession of the electrons with the applied magnetic field. The delicate interplay between the paramagnetic and diamagnetic Zeeman terms and their balance with the Coulombic interactions of the electrons leads to quite complex ground state chemistry for magnetic fields on the order of 1*B*_0_ in strength.

The situation for excited states in magnetic fields is less well studied, though recent works have begun to explore this area.^6,10,43,74^ In general, it is expected that excited states may be more susceptible to the effects of strong magnetic fields due to their typically more diffuse electronic structure. Extra transitions corresponding to the lifting of degeneracies appear in spectra, and in addition, magnetic fields may break spatial symmetries, meaning that symmetry forbidden transitions become allowed. Such effects will naturally depend on the orientation of the molecular system relative to the field. This means that the spectra of even simple molecular systems can become significantly more complicated in a magnetic field.

To investigate these effects, we consider the N_2_ and H_2_O molecules subject to external magnetic fields applied in different orientations at the cTPSSrsh/6-311++G** level, for which the zero-field spectra were analyzed in Section 3.2. The calculations were performed in magnetic fields parallel (*B*_∥_), at 45° (*B*_45°_), and perpendicular (*B*_⊥_) to the internuclear axis of N_2_ with field strengths ranging from 0.0 to 0.25*B*_0_. For H_2_O, which was placed in the *yz* plane (molecular plane) with the *z* axis as the *C*_2_ symmetry axis, the magnetic field was applied parallel (*B_z_*) and perpendicular (*B_x_*) to the molecular plane with field strengths ranging from 0.0 to 0.5*B*_0_.

#### 3.3.1 The N_2_ Molecule

For a homonuclear diatomic molecule such as N_2_, the presence of a magnetic field results in the symmetry of the electronic structure being lowered from *D*_∞*h*_. The extent to which the symmetry is lowered depends on the orientation of the magnetic field relative to the internuclear axis. For magnetic fields aligned parallel to the internuclear axis, the symmetry is reduced to *C*_∞*h*_, preserving only the *C*_∞_, *i*, and *σ_h_* (mirror plane perpendicular to the internuclear axis) symmetry elements. For magnetic fields aligned perpendicular to the internuclear axis, the symmetry is reduced to *C*_2*h*_, having a *C*_2_ axis perpendicular to bond axis and along the field direction, a *σ_h_* in the plane perpendicular to the axis containing the two nuclei and a center of inversion *i*. For all other orientations of the magnetic field, the symmetry is reduced to *C_i_*, with only the center of inversion preserved.^75,76^ The molecular orbitals then belong to the irreducible representations of these point groups, rather than those of the *D*_∞*h*_ point group. In Table 4, the molecular orbital labels are given for N_2_ in each of these point groups in order of the zero-field ground state configuration predicted at the cTPSSrsh/6-311++G** level, as discussed in Section 3.2, for the first 26 orbitals.

**Table 4:** The First 26 Molecular Orbitals in N_2_ for cTPSSrsh/6-311++G** Calculations, Labeled According to the Point Group *D*_∞*h*_, and the Subgroups *C*_∞*h*_, *C*_2*h*_, and *C_i_*^a^

orb. no.	*D*_∞*h*_	*C*_∞*h*_	*C*_2*h*_	*C_i_*
26	7*σ_u_*	7*σ_u_*	7*a_u_*	13*a_u_*
25	6*σ_u_*	6*σ_u_*	6*a_u_*	12*a_u_*
24	7*σ_g_*	7*σ_g_*	7*a_g_*	13*a_g_*
23	3*π*_*g*+1_	3*π*_*g*+1_	6*b_g_*	12*a_g_*
22	3*π*_*g*–1_	3*π*_*g*–1_	5*b_g_*	11*a_g_*
21	3*π*_*u*+1_	3*π*_*u*+1_	6*b_u_*	11*a_u_*
20	3*π*_*u*–1_	3*π*_*u*–1_	5*b_u_*	10*a_u_*
19	6*σ_g_*	6*σ_g_*	6*a_g_*	10*a_g_*
18	5*σ_u_*	5*σ_u_*	5*a_u_*	9*a_u_*
17	4*σ_u_*	4*σ_u_*	4*a_u_*	8*a_u_*
16	2*π*_*g*+1_	2*π*_*g*+1_	4*b_g_*	9*a_g_*
15	2*π*_*g*–1_	2*π*_*g*–1_	3*b_g_*	8*a_g_*
14	5*σ_g_*	5*σ_g_*	5*a_g_*	7*a_g_*
13	2*π*_*u*+1_	2*π*_*u*+1_	4*b_u_*	7*a_u_*
12	2*π*_*u*–1_	2*π*_*u*–1_	3*b_u_*	6*a_u_*
11	3*σ_u_*	3*σ_u_*	3*a_u_*	5*a_u_*
10	4*σ_g_*	4*σ_g_*	4*a_g_*	6*a_g_*
9	1*π*_*g*+1_	1*π*_*g*+1_	2*b_g_*	5*a_g_*
8	1*π*_*g*–1_	1*π*_*g*–1_	1*b_g_*	4*a_g_*
7	3*σ_g_*	3*σ_g_*	3*a_g_*	3*a_g_*
6	1*π*_*u*+1_	1*π*_*u*+1_	2*b_u_*	4*a_u_*
5	1*π*_*u*–1_	1*π*_*u*–1_	1*b_u_*	3*a_u_*
4	2*σ_u_*	2*σ_u_*	2*a_u_*	2*a_u_*
3	2*σ_g_*	2*σ_g_*	2*a_g_*	2*a_g_*
2	1*σ_u_*	1*σ_u_*	1*a_u_*	1*a_u_*
1	1*σ_g_*	1*σ_g_*	1*a_g_*	1*a_g_*

a In the absence of a magnetic field, the ±1 components of the *π* orbitals are degenerate.

As demonstrated in Section 3.2, the MO pair decomposition analysis^32,38^ is very useful for assigning the dominant orbital contributions for peaks in the electronic absorption spectra. A significant complicating factor in the presence of a magnetic field is that the degeneracies of the *π* orbitals in Table 4 are lifted, the splitting depending on both the strength and the orientation of the applied field. The energies of the *σ* orbitals also change as a result of changes in the electronic structure under an applied field. As a result, the orbital energy spectrum may significantly reorder for |**B**| > 0. Noting that the non-degeneracy of the *π* orbitals in the presence of a magnetic field allows their separate identification, molecular orbitals are first evaluated in the presence of a small but non-zero magnetic field **B** in the direction chosen. These can be related to molecular orbitals obtained with a magnetic field of slightly higher or lower strength applied in the same direction, **B** ± *δ***B**, by computing the overlaps
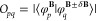
35and selecting the index *q* for the largest overlap with each initial orbital *p*. Considering the orbitals evaluated at **B** → **B** ± *δ***B**, a similar procedure can be applied to track the molecular orbital energies and assignments as a function of magnetic field across the entire range of interest.

**Figure 3:**
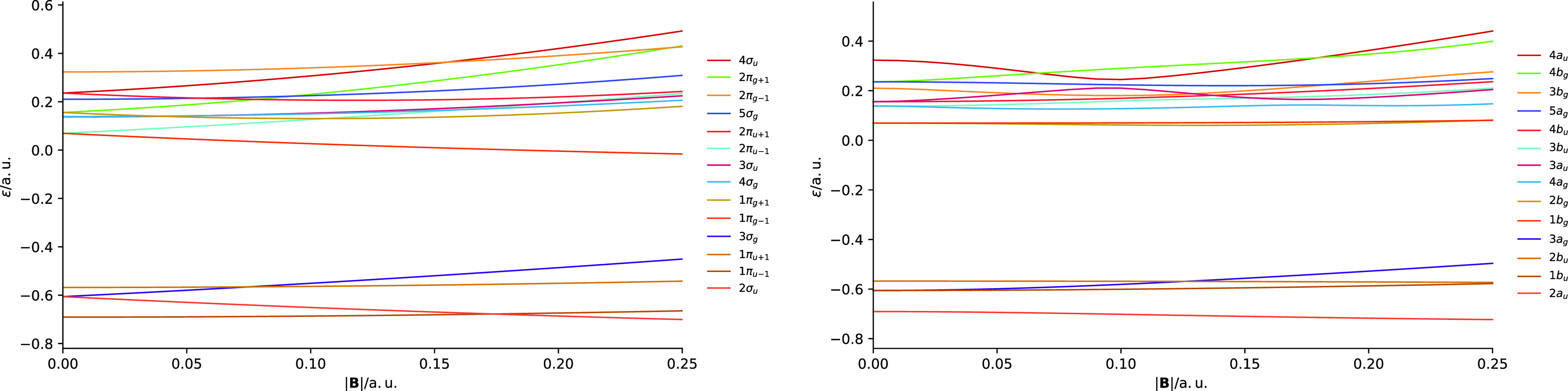
Molecular orbital energies as a function of magnetic fields oriented parallel (left) and perpendicular (right) to the internuclear axis of the N_2_ molecule. All calculations at the cTPSSrsh/6-311++G** level.

Figure 3 shows the orbital energies as a function of magnetic field for parallel (left panel) and perpendicular (right panel) orientations. The parallel orientation (*C*_∞*h*_ symmetry) shows strong splitting of the *π*_*u*/*g*±1_ orbital degeneracies. This has the consequence that the orbitals reorder but also that, at some field strengths, accidental degeneracies between *π* and *σ* orbitals are present. A case in point is the splitting of the 1*π*_*u*–1_/1*π*_*u*+1_ orbitals. The 1*π*_*u*–1_ orbital lowers in energy, becoming near degenerate with the 2*σ_u_* orbital at fields close to 0.15*B*_0_. The 1*π*_*u*+1_ orbital increases in energy becoming near degenerate with the 3*σ_g_* orbital around 0.08*B*_0_. Similar near degeneracies can be observed for the virtual orbitals in the left-hand panel of Figure 3. At stronger fields, the orbitals again become reasonably well separated, though re-ordered compared with their zero-field counterparts.

In a perpendicular field (*C*_2*h*_ symmetry), shown in the right panel of Figure 3, the orbital energies display more subtle variation. At this orientation, the change in energy due to the orbital paramagnetic terms in the Hamiltonian of eq 34 is minimized. The 1*b_u_* and 2*b_u_* orbitals remain close in energy throughout the field range 0.00–0.25*B*_0_. Their degeneracy is however lifted due to the quadratic term in eq 34. This term dominates at very high fields but has a relatively subtle effect at lower fields. In the perpendicular orientation, these more subtle effects are revealed. The degeneracy of the *b_u_* and *b_g_* orbitals are lifted slightly due to the compression of the electronic structure, which is slightly more pronounced in the directions perpendicular to the field than along it. It is also notable that the orbitals with the *a_g_* symmetry tend to increase slightly in energy with field, whilst those of the *a_u_* symmetry decrease slightly with field. This effect arises because the magnetic field causes the atomic orbitals to contract toward the nuclei, reducing their overlap, which leads to an increase in energy for bonding combinations (reduction of favorable overlap) and a decrease in energy for anti-bonding combinations (reduction of unfavorable overlap).

**Figure 4:**
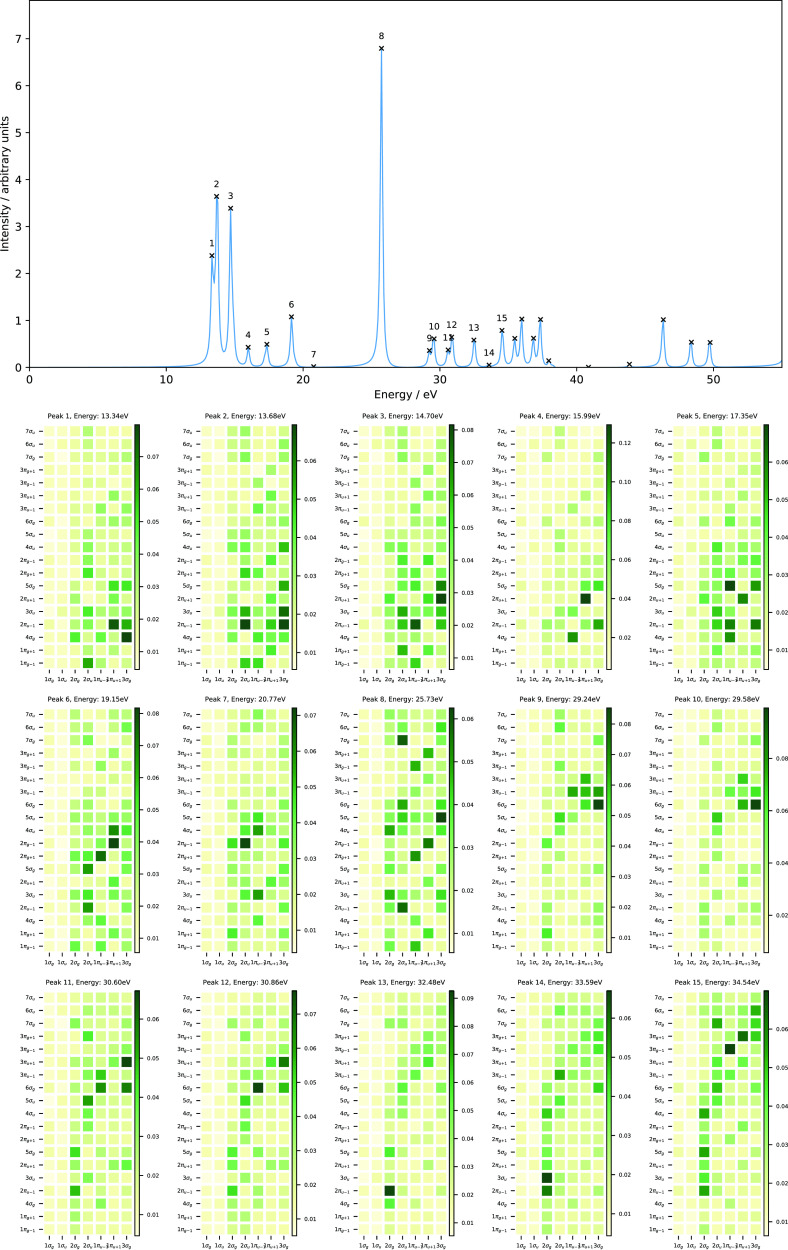
Electronic absorption spectrum and MO pair decomposition analysis for the N_2_ molecule in a parallel magnetic field, *B*_∥_ = 0.05*B*_0_, computed using the cTPSSrsh functional and 6-311++G** basis set.

**Figure 5:**
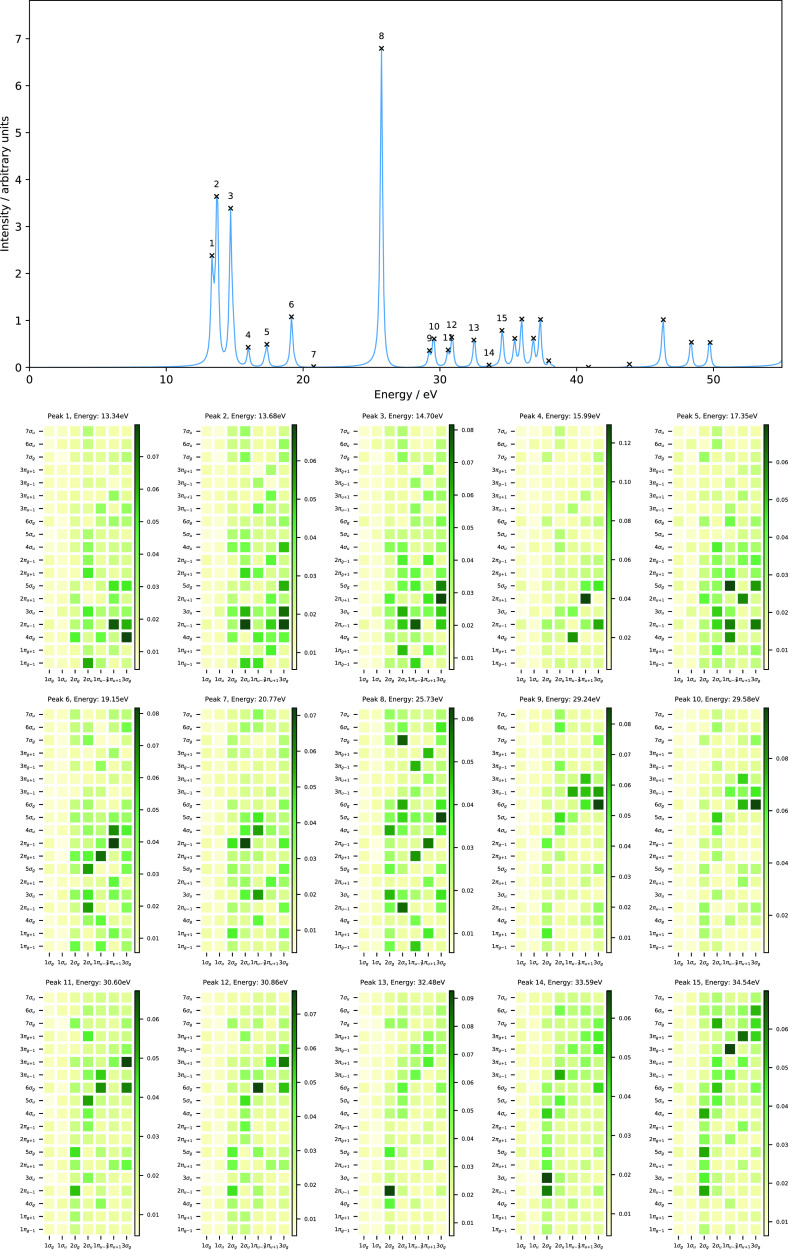
Electronic absorption spectrum and MO pair decomposition analysis for the N_2_ molecule in a parallel magnetic field, *B*_∥_ = 0.15*B*_0_, computed using the cTPSSrsh functional and 6-311++G** basis set.

**Figure 6:**
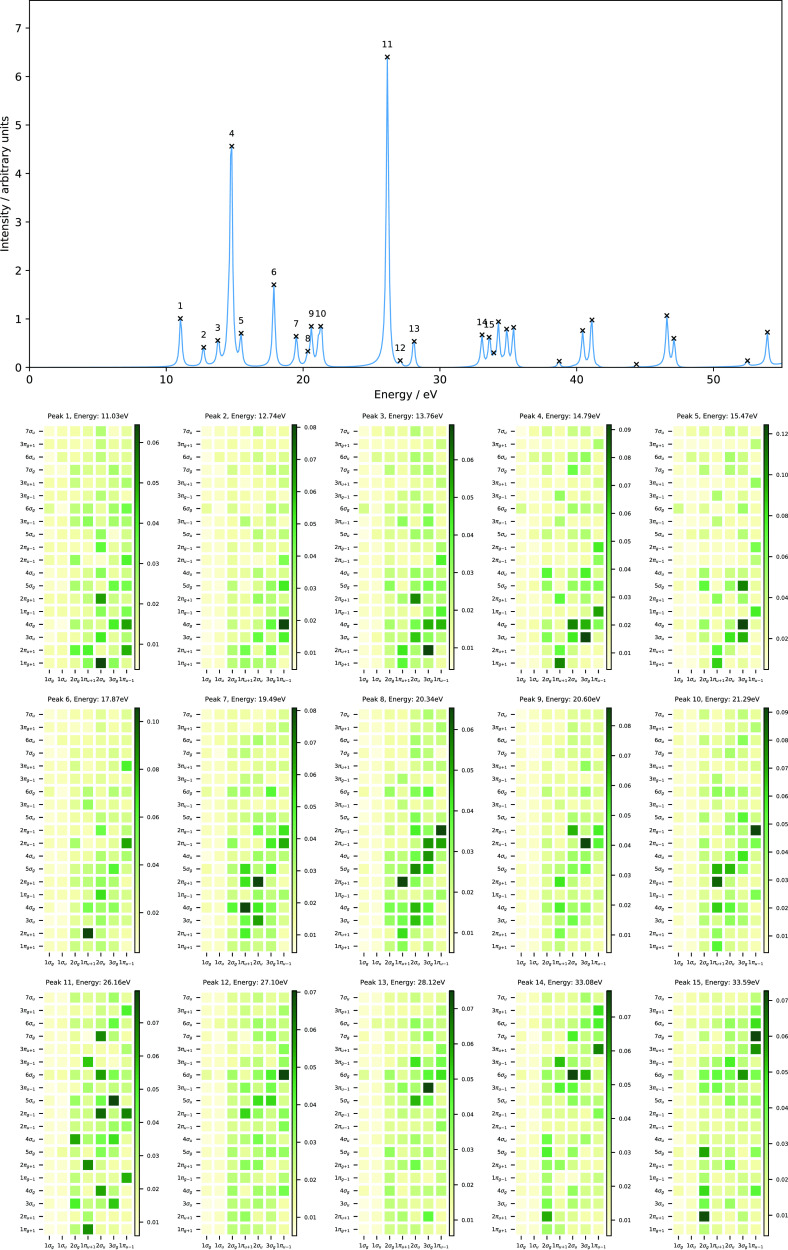
Electronic absorption spectrum and MO pair decomposition analysis for the N_2_ molecule in a parallel magnetic field, *B*_∥_ = 0.25*B*_0_, computed using the cTPSSrsh functional and 6-311++G** basis set.

We now consider how the absorption spectra evolve as a function of magnetic field when the field vector is parallel to the internuclear axis. In this case, the reduction in symmetry is lowest, with the point group lowered from *D*_∞*h*_ to *C*_∞*h*_, but the splitting of the *π* orbitals is most pronounced, leading to significant changes in the spectra. These are shown in Figure 4–6 for *B*_∥_ = 0.05, 0.15, and 0.25*B*_0_, respectively. These may be compared with the *B* = 0.0*B*_0_ spectrum in Figure 1.

The first 10 peaks for each parallel field strength *B*_∥_ = 0.0, 0.05, 0.15, and 0.25*B*_0_ are shown in Table 5. The dominant orbital characters, from the MO pair decomposition analysis, for each transition are given along with the excitation energy. The orbital characters at zero field are color coded; at zero field, one color corresponds to each peak, and additional contributions at higher fields are given in black. This information summarizes how the spectra change as a function of field. For example, at zero field, the first peak at 13.68 eV has a dominant contribution from the transition 3*σ_g_*→3*σ_u_*, colored red in Table 5. The second peak also has some character of 3*σ_g_*→3*σ_u_*, along with the (degenerate) 3*σ_g_*→2*π*_*u*±1_. The mixed characters of many of the peaks in the spectra reflect the high spectral density of N_2_. In the presence of a field, the degeneracy of the 2*π*_*u*–1_ and 2*π*_*u*+1_ orbitals is lifted (see Figure 3). Accordingly, we see that the first peak with *B*_∥_ = 0.05*B*_0_ at 13.34 eV has the 3*σ_g_*→4*σ_g_* character, whilst the second and third peaks have the 3*σ_g_*→2*π*_*u*–1_ and 3*σ_g_*→2*π*_*u*+1_ character, respectively. In addition, peak 2 retains the 3*σ_g_*→3*σ_u_* character since the *σ* orbitals are more weakly affected by the field.

**Table 5:**
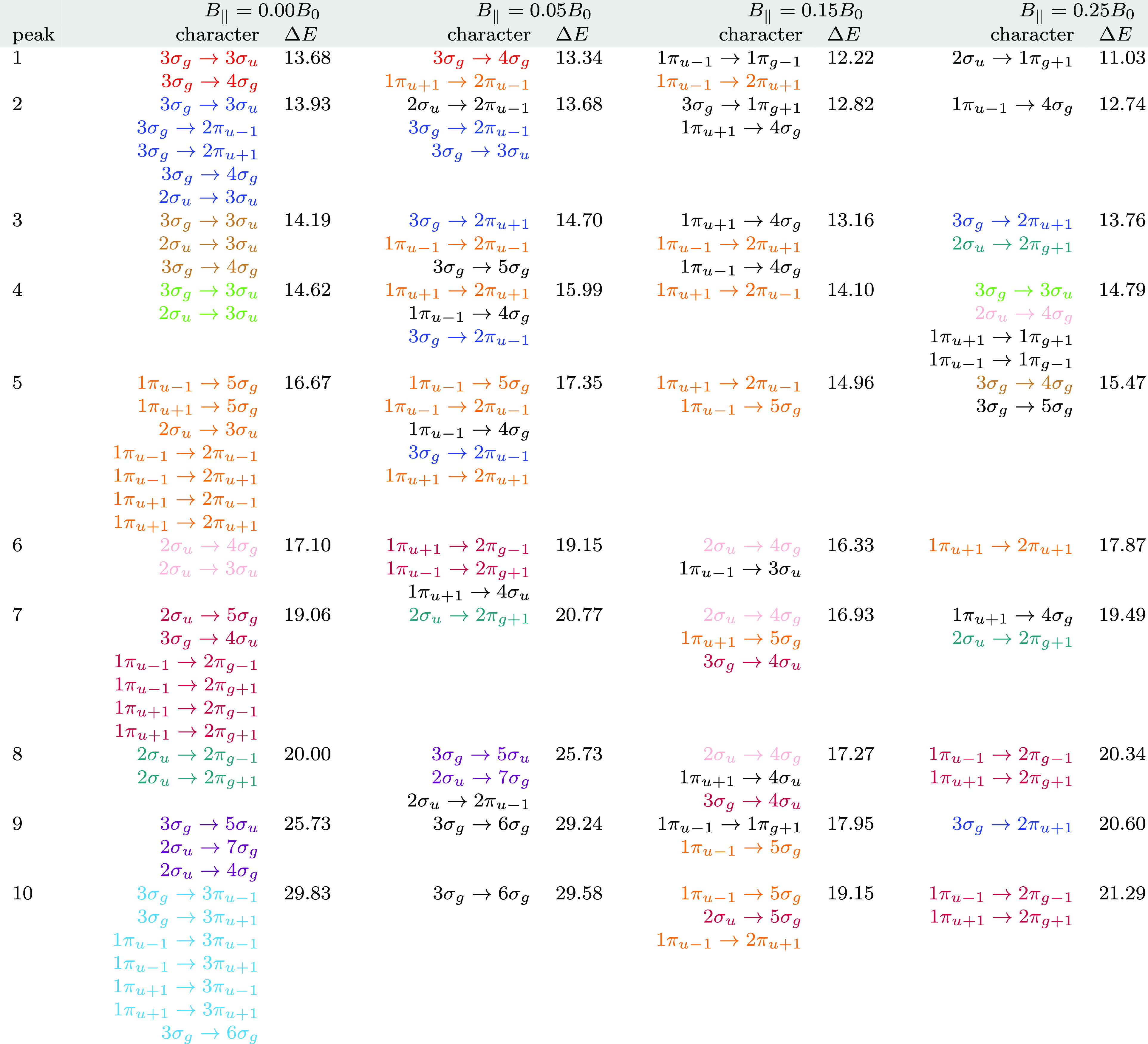
Excitation Energies (Δ*E*, in eV) and Dominant Orbital Characters from the MO Pair Decomposition Analysis for N_2_ in a Magnetic Field Parallel to the Internuclear Axis^a^

a All calculations at the cTPSSrsh/6-311++G** level.

In general, it is clear that the transitions have complex character with many contributions, and similar conclusions are obtained from linear response calculations at the RPA level. In weaker fields *B*_∥_ = 0.05 and 0.15*B*_0_, this complexity relative to zero field is amplified by the magnetic field since, although orbital degeneracies are lifted, the field also reduces the symmetry and allows for further mixing. At the strongest field considered here, it is clear that the peaks in the absorption spectra start to become more distinct and; as a result, the MO pair decompositions show that the transitions are more readily assigned to individual orbital pairs, as shown in Figure 6.

In the parallel orientation, where the most symmetry is preserved, the evolution of the spectra can be understood by following the character of the transitions, as shown in Table 5. A few general observations can be made; *σ*→*σ* transitions are only weakly affected by the magnetic field, as expected from the orbital energies in Figure 3. In contrast, the *σ*→*π* transitions are strongly affected. This mixture of weakly and strongly affected transitions leads to significant re-structuring of the spectra as a function of field.

**Figure 7:**
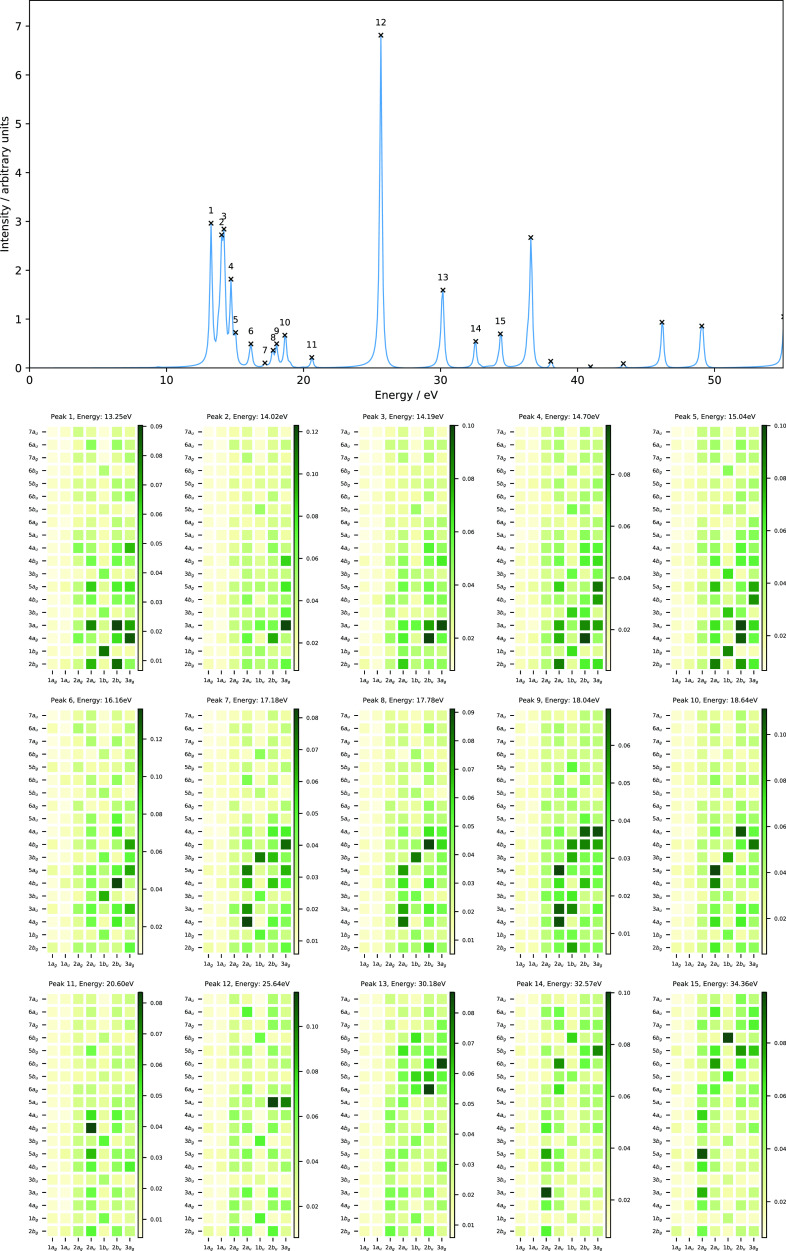
Electronic absorption spectrum and MO pair decomposition analysis for the N_2_ molecule in a perpendicular magnetic field, *B*_⊥_ = 0.05*B*_0_, computed using the cTPSSrsh functional and 6-311++G** basis set.

**Figure 8:**
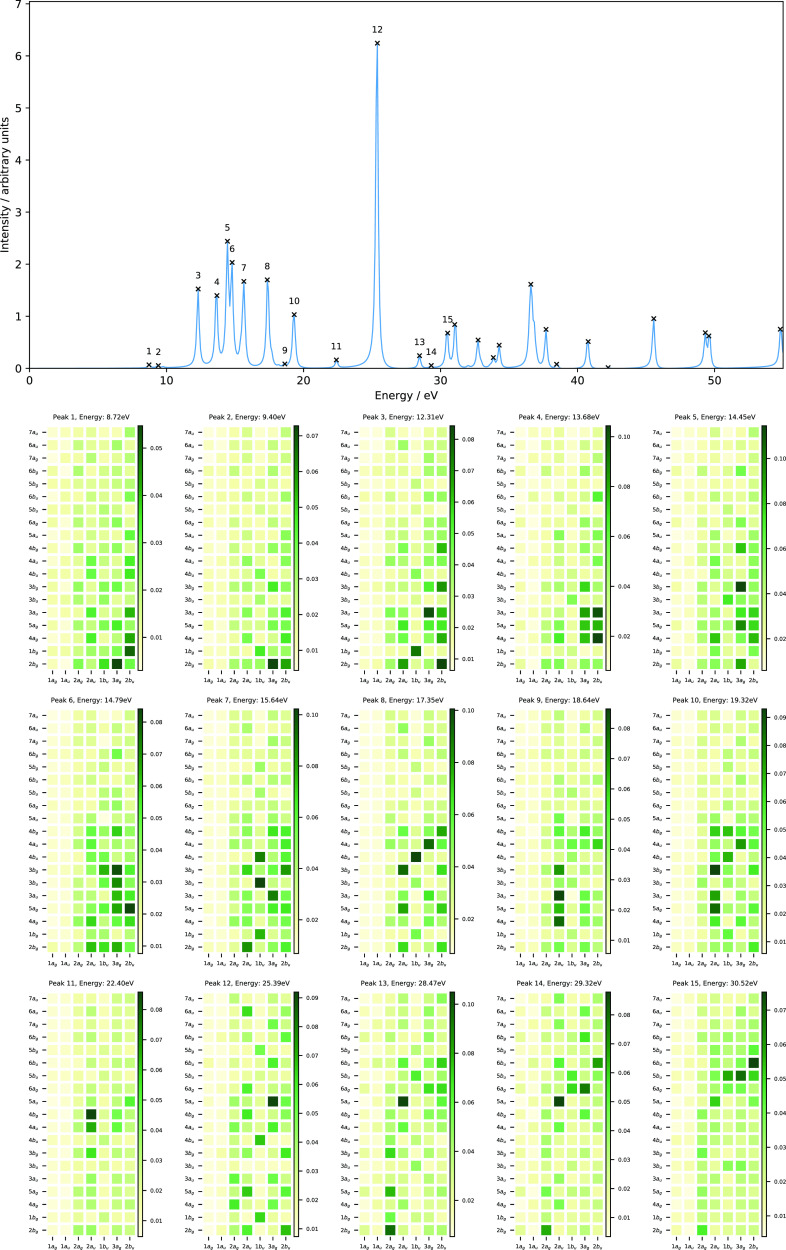
Electronic absorption spectrum and MO pair decomposition analysis for the N_2_ molecule in a perpendicular magnetic field, *B*_⊥_ = 0.15*B*_0_, computed using the cTPSSrsh functional and 6-311++G** basis set.

**Figure 9:**
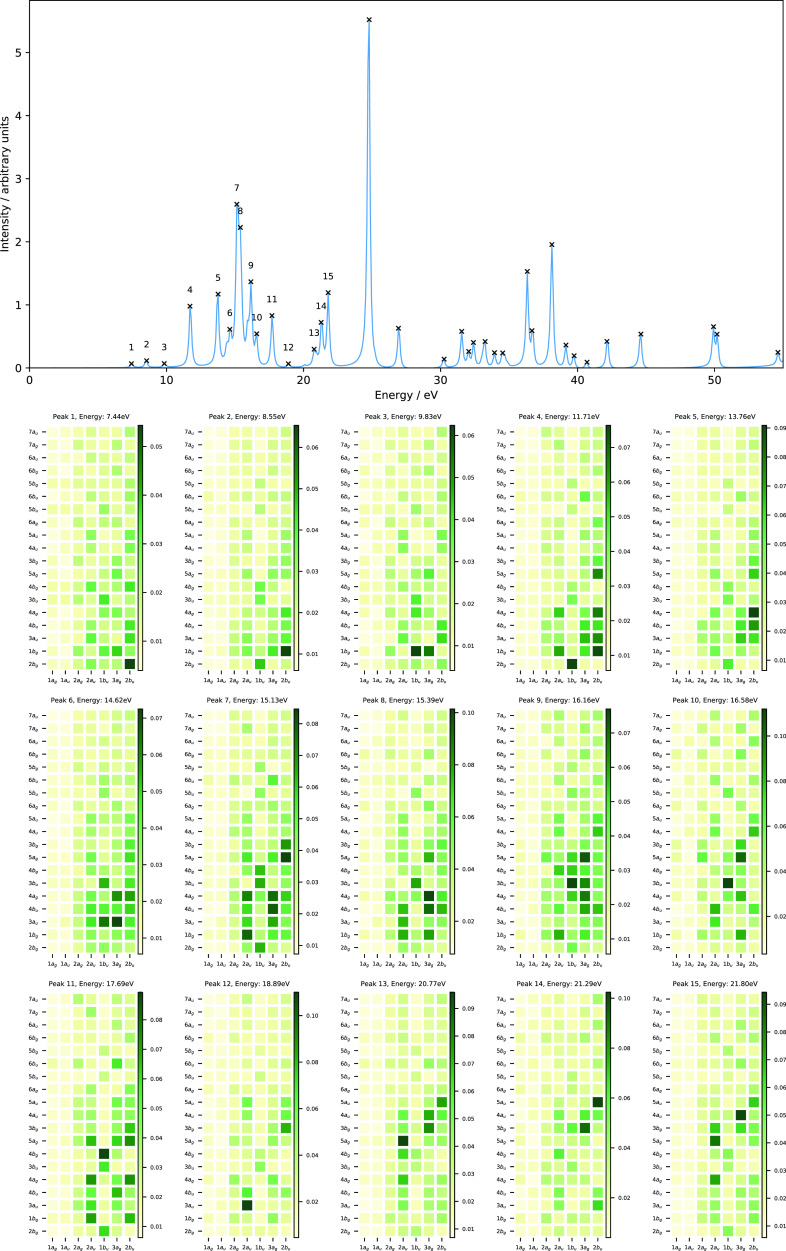
Electronic absorption spectrum and MO pair decomposition analysis for the N_2_ molecule in a perpendicular magnetic field, *B*_⊥_ = 0.25*B*_0_, computed using the cTPSSrsh functional and 6-311++G** basis set.

The excitation energies for N_2_ in a perpendicular magnetic field *B*_⊥_ = 0.05, 0.15, and 0.25*B*_0_ are shown in Table 6. The symmetry in this orientation is reduced to *C*_2*h*_, and the first 10 peaks in the electronic absorption spectrum are shown with their dominant orbital character, assigned according to the irreducible representations of the *C*_2*h*_ point group. At *B*_⊥_ = 0.05*B*_0_, the spectrum in Figure 7 bears close resemblance to the zero-field spectrum in Figure 1. However, the orbital energies are affected much less as a function of magnetic field than when the field is perpendicular to the internuclear axis, as shown in the right panel of Figure 3. As a result, the mixed character of the peaks is even more pronounced at low field compared with the parallel orientation. A notable feature at stronger fields, *B*_⊥_ = 0.15 and 0.25*B*_0_, is that transitions at lower energies gain significant intensity, notably those at 8.72 and 9.04 eV for *B*_⊥_ = 0.15*B*_0_ and 7.44, 8.55, and 9.83 eV at *B*_⊥_ = 0.25*B*_0_, giving rise to the extra peaks in Figures 8 and 9. These transitions would be symmetry forbidden in the absence of a magnetic field. It is also notable that, at *B*_⊥_ = 0.25*B*_0_, as the field begins to lift the orbital degeneracies significantly, each peak is more dominated by a single transition (see Figures 3 and 9).

**Table 6:**
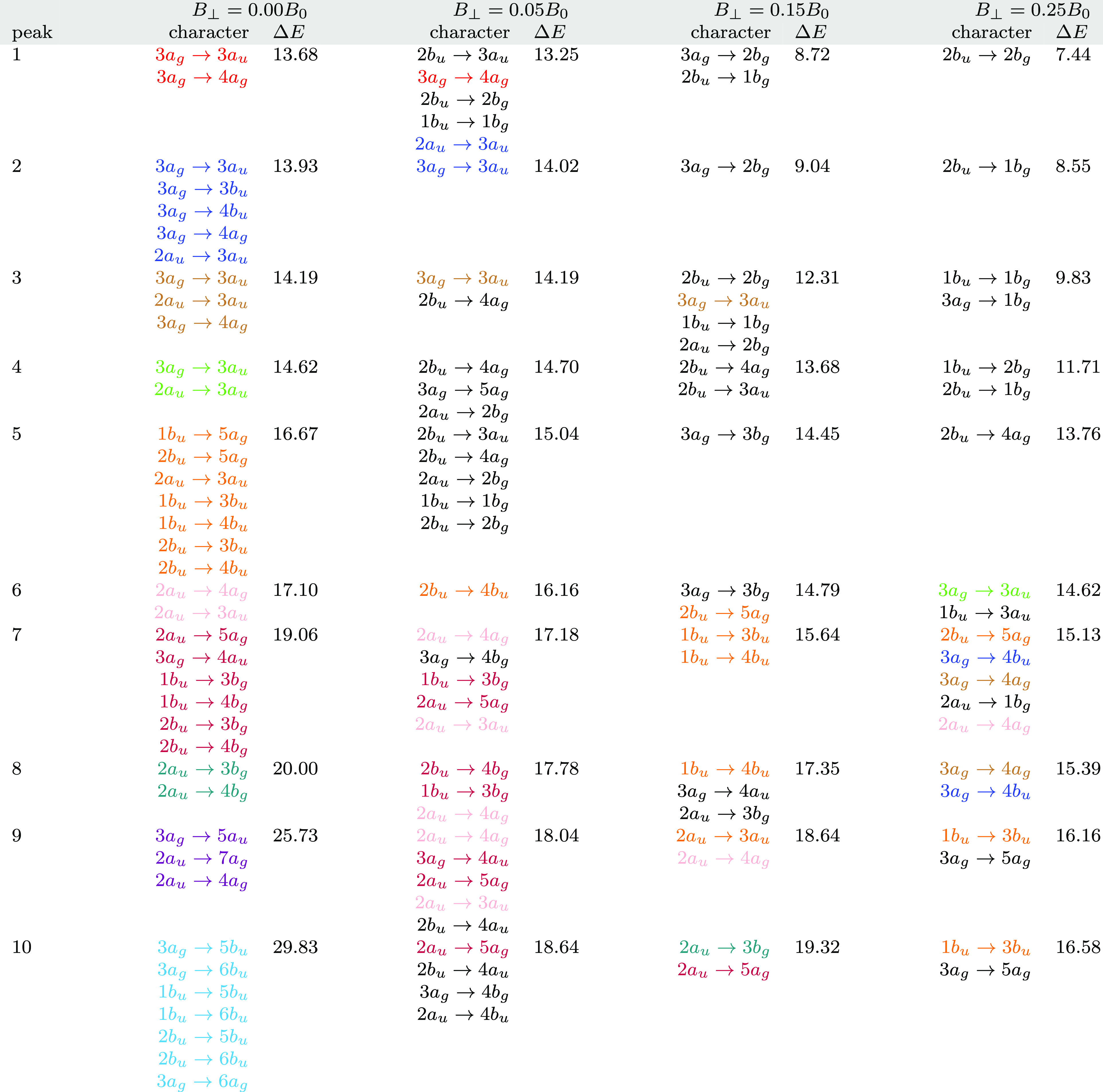
Excitation Energies (Δ*E*, in eV) and Dominant Orbital Characters from the MO Pair Decomposition Analysis for N_2_ in a Magnetic Field Perpendicular to the Internuclear Axis^a^

a All calculations at the cTPSSrsh/6-311++G** level.

At all other orientations, the symmetry is reduced to *C_i_*. As an example we consider a field oriented at 45° to the internuclear axis. The excitation energies and their orbital characters are presented in Table S2 in the Supporting Information, labeled according to the irreducible representations of the *C_i_* point group. At *B*_45°_ = 0.05*B*_0_, the spectrum in Figure S4 in the Supporting Information resembles that for *B*_∥_ = 0.05*B*_0_ closely. This reflects the similarity of the behavior of the orbital energies as a function of magnetic field shown in Figure S3 in the Supporting Information to those in the parallel orientation (see Figure 3). A general observation is that, for a given strength of magnetic field, degeneracies are lifted rapidly as the molecule moves away from the perpendicular orientation, meaning that, for the majority of orientations, the orbital energies and spectra more closely resemble the parallel orientation than the perpendicular one. Of course, the reduced symmetry can still lead to additional transitions that would be forbidden at zero field.

The example of N_2_ at the cTPSSrsh/6-311++G** level is a case that illustrates many of the complexities that arise due to the presence of a magnetic field. By combining knowledge of the orbital spectrum as a function of magnetic field (within the relevant reduced symmetries) with the MO pair decomposition analysis of the spectra, it is possible to understand in detail the evolution of the spectra as a function of field. Re-ordering of the peaks is commonplace as are accidental degeneracies, and these tools give the essential insight to unravel the complexity of the resulting spectra. At zero field, the N_2_ molecule has a high spectral density with complex transitions consisting of contributions from different MO pairs. In the presence of a field, the symmetry is lowered and many degeneracies are lifted, resulting in spectra with many more visible peaks. However, in many cases, these peaks become more distinct and more dominated by individual (or fewer) MO pair contributions as the field strength is increased.

#### 3.3.2 The H_2_O Molecule

We now consider a simple polyatomic molecule with lower symmetry. In the presence of a magnetic field, the *C*_2*v*_ symmetry of the H_2_O molecule, which is placed in the *yz* plane (molecular plane) with the *z* axis as the *C*_2_ symmetry axis, is no longer preserved. The symmetry of H_2_O depends on the orientation with respect to the magnetic field. In a magnetic field parallel to the molecular plane (*B_z_*), the symmetry of H_2_O is lowered to the *C*_2_ symmetry, in which the magnetic field is parallel to the *C*_2_ symmetry axis. The symmetry of H_2_O is further lowered to the *C_s_* symmetry when the magnetic field is applied perpendicular to the molecular plane (*B_x_*). In Table 7, the first 16 molecular orbitals labels are given for each of these point groups in order of the zero-field ground state configuration predicted at the cTPSSrsh/6-311++G** level, as discussed in Section 3.2.

**Table 7:** The First 16 Molecular Orbitals in H_2_O for cTPSSrsh/6-311++G** Calculations, Labeled According to the Point Group *C*_2*v*_, and the Subgroups *C*_2_ and *C_s_*

orb. no.	*C*_2*v*_	*C*_2_	*C_s_*
16	9*a*_1_	9*a*	14*a*^′^
15	8*a*_1_	8*a*	13*a*^′^
14	5*b*_2_	7*b*	12*a*^′^
13	7*a*_1_	7*a*	11*a*^′^
12	4*b*_2_	6*b*	10*a*^′^
11	6*a*_1_	6*a*	9*a*^′^
10	3*b*_2_	5*b*	8*a*^′^
9	2*b*_1_	4*b*	2*a*^″^
8	5*a*_1_	5*a*	7*a*^′^
7	2*b*_2_	3*b*	6*a*^′^
6	4*a*_1_	4*a*	5*a*^′^
5	1*b*_1_	2*b*	1*a*^″^
4	3*a*_1_	3*a*	4*a*^′^
3	1*b*_2_	1*b*	3*a*^′^
2	2*a*_1_	2*a*	2*a*^′^
1	1*a*_1_	1*a*	1*a*^′^

**Figure 10:**
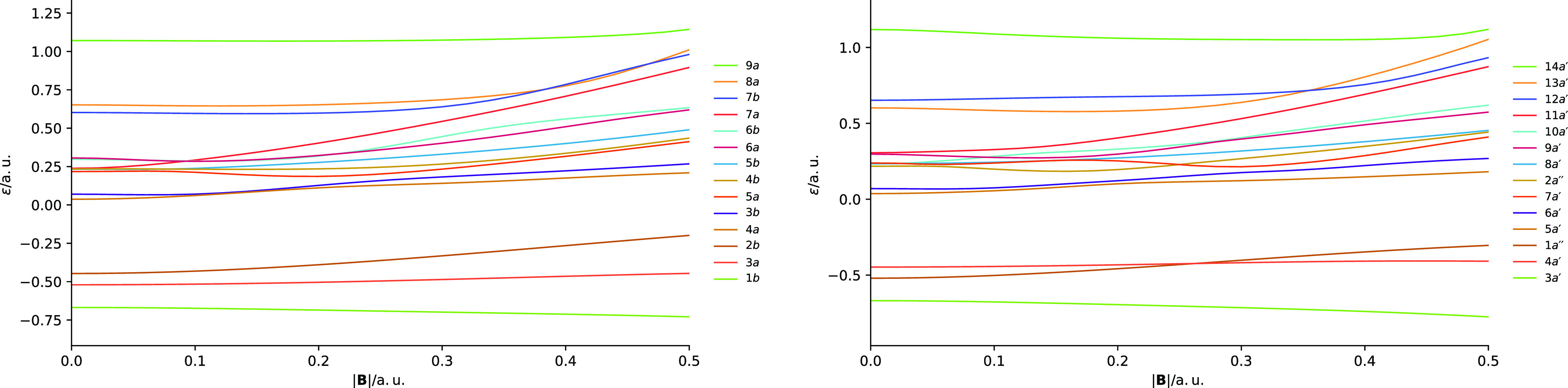
Molecular orbital energies of H_2_O as a function of magnetic fields parallel (left) and perpendicular (right) to the molecular plane, computed using the cTPSSrsh functional and 6-311++G** basis set. The H_2_O molecule is placed in the *yz* plane (molecular plane) with the *z* axis as the *C*_2_ symmetry axis.

Figure 10 shows the evolution of the orbital energies as a function of magnetic field strength parallel (left panel) and perpendicular (right panel) to the molecular plane. The valence orbitals at zero field comprise the 1*b*_2_, 3*a*_1_, 1*b*_1_, 4*a*_1_, and 2*b*_2_ orbitals (see Section 3.2). They correspond to the 1*b*, 3*a*, 2*b*, 4*a*, and 3*b* orbitals for a magnetic field parallel to the molecular plane and the 3*a*^′^, 4*a*^′^, 1*a*^″^, 5*a*^′^, and 6*a*^′^ orbitals for a magnetic field perpendicular to the molecular plane (see Table 7).

**Figure 11:**
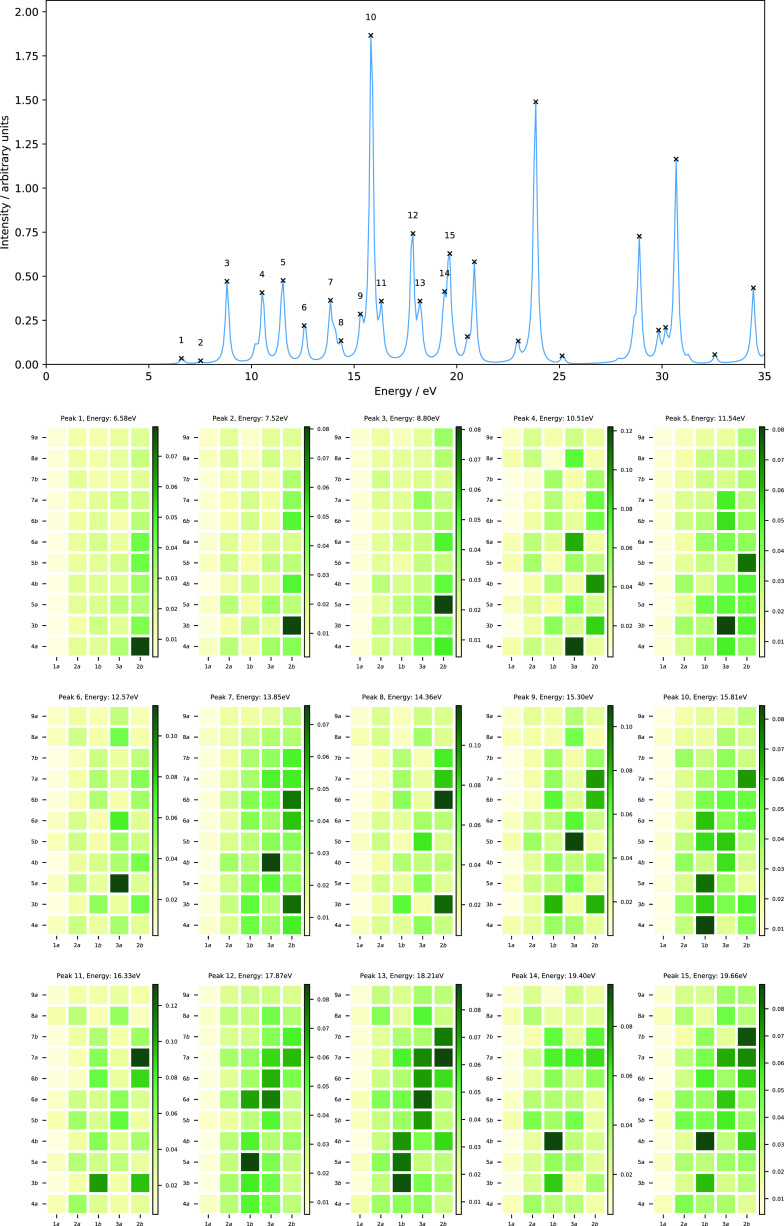
Electronic absorption spectrum and MO pair decomposition analysis for the H_2_O molecule in a magnetic field parallel to the molecular plane, *B_z_* = 0.25*B*_0_, computed using the cTPSSrsh functional and 6-311++G** basis set. The H_2_O molecule is placed in the *yz* plane (molecular plane) with the *z* axis as the *C*_2_ symmetry axis.

**Figure 12:**
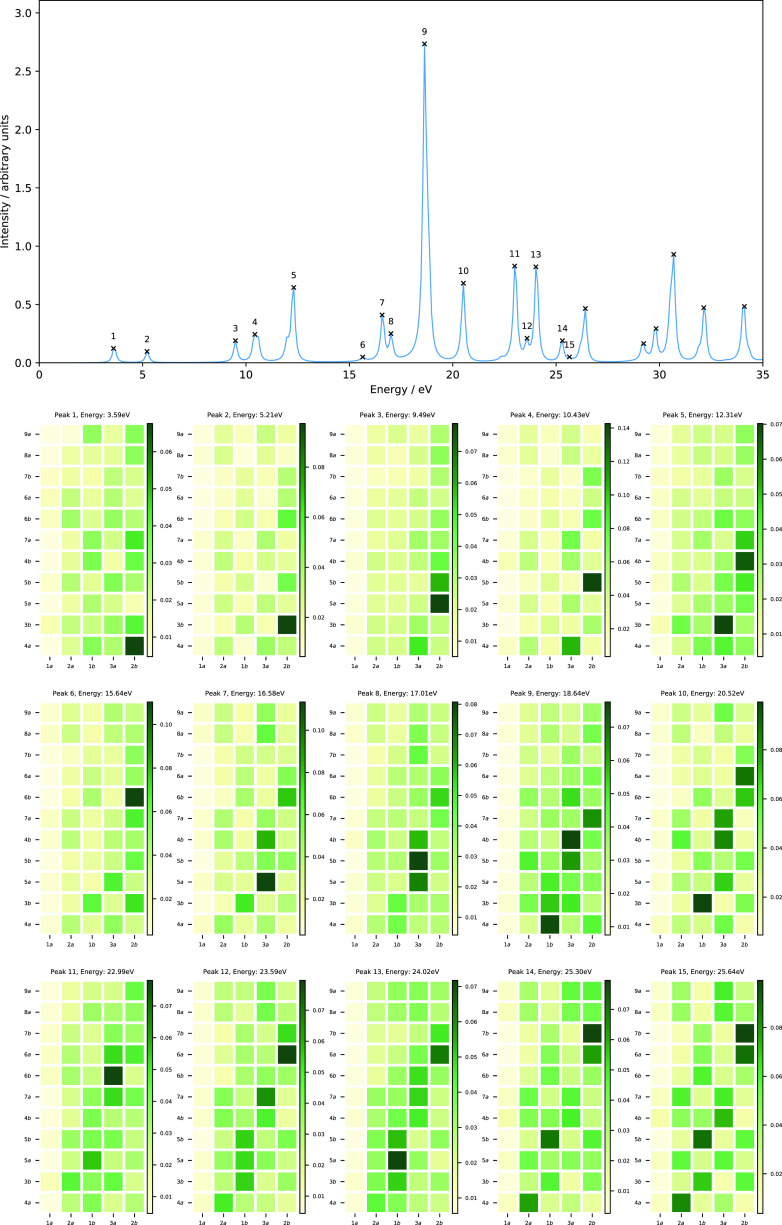
Electronic absorption spectrum and MO pair decomposition analysis for the H_2_O molecule in a magnetic field parallel to the molecular plane, *B_z_* = 0.50*B*_0_, computed using the cTPSSrsh functional and 6-311++G** basis set. The H_2_O molecule is placed in the *yz* plane (molecular plane) with the *z* axis as the *C*_2_ symmetry axis.

In the parallel case (*C*_2_ symmetry), the valence orbitals evolve in a rather simple manner. The energies of these orbitals are slightly increased in strong magnetic fields except the HOMO-2 (1*b* orbital). The first peak which involves a transition from the HOMO → LUMO (2*b*→4*a*) at both field strengths *B_z_* = 0.25 and 0.50*B*_0_ is red-shifted by about 0.86 and 3.85 eV, respectively (see Figures 11 and 12 and Table 8). This transition describes an excitation from the lone pair of the oxygen atom to the O–H anti-bonding orbital.

**Table 8:**
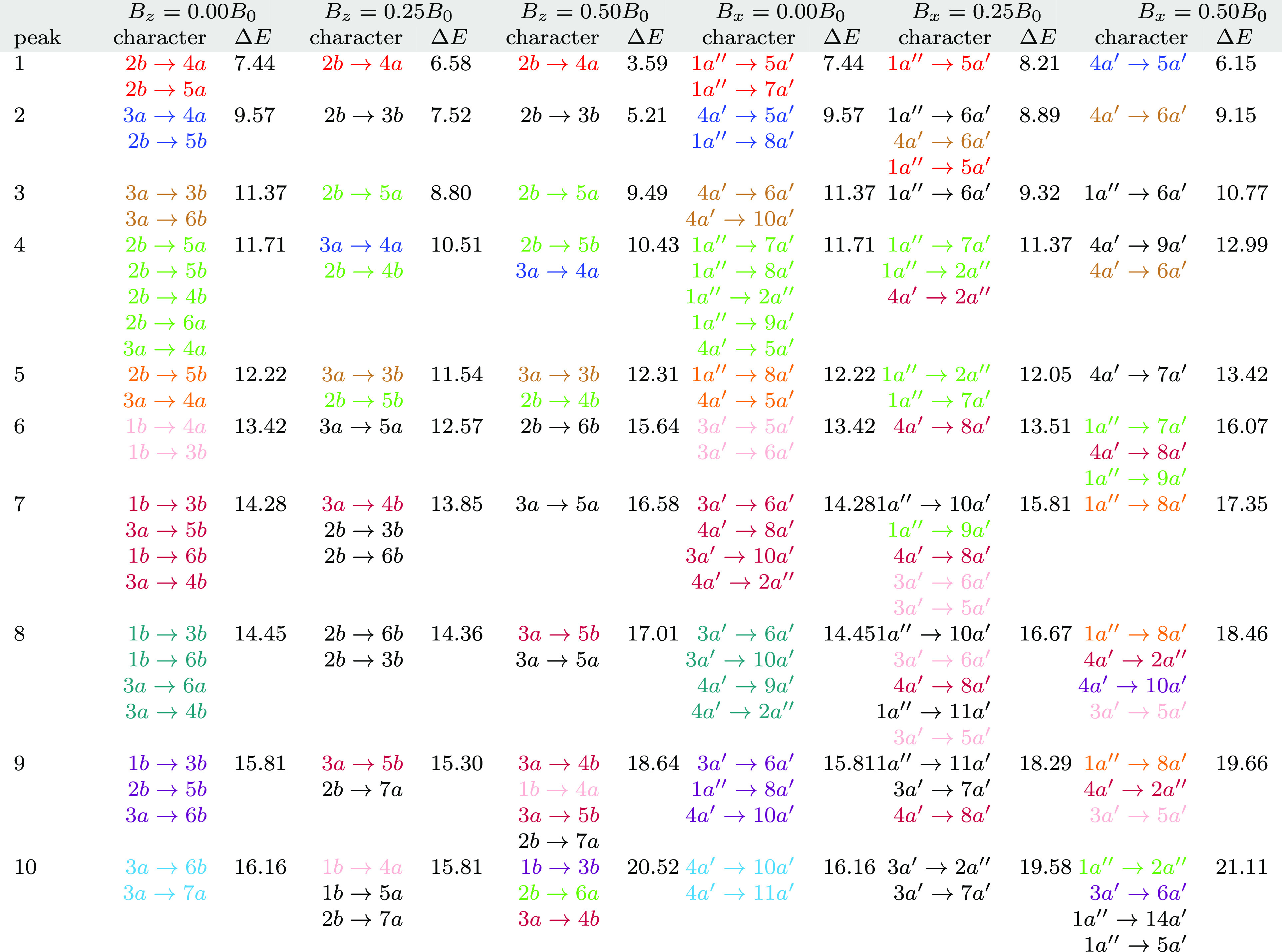
Excitation Energies (Δ*E*, in eV) and Dominant Orbital Characters from the MO Pair Decomposition Analysis for H_2_O in Magnetic Fields Parallel (*B_z_*) and Perpendicular (*B_x_*) to the Molecular Plane^a^

a The H_2_O molecule is placed in the *yz* plane (molecular plane) with the *z* axis as the *C*_2_ symmetry axis. All calculations at the cTPSSrsh/6-311++G** level.

At *B_z_* = 0.25*B*_0_, the second peak corresponds to the transition from HOMO → LUMO+1 (2*b*→3*b*) and is analogous to a 1*b*_1_→2*b*_2_ transition, which would be symmetry forbidden at zero field. The third peak at 8.80 eV is dominated by one-electron promotion from the HOMO → LUMO+2 (2*b*→5*a*) and is similar to peak 4 in the zero-field spectrum at 11.71 eV but with less mixing. The fourth peak is dominated by a transition with the 3*a*→4*a* character, corresponding to peak 2 at zero field and is blue-shifted from the zero-field value by 0.94 eV. The fifth peak shows a mixed character of 3*a*→3*b* and 2*b*→5*b* transitions, in which the latter is analogous to a forbidden transition at zero field (1*b*_1_→3*b*_2_).

As we increase the magnetic field strength to *B_z_* = 0.50*B*_0_, one noticeable effect is the red-shift of the two lowest peaks, i.e., from 6.58 and 7.52 eV at *B_z_* = 0.25*B*_0_ to 3.59 and 5.21 eV at *B_z_* = 0.50*B*_0_. As a consequence, peaks 2 and 3 are well separated at *B_z_* = 0.50*B*_0_. However, the MO pairs involved in these transitions are practically the same as those involved at *B_z_* = 0.25*B*_0_ (see Table 8). The excitation energies of peaks 3 and 5, on the other hand, are blue-shifted by about 0.69 and 0.77 eV, respectively. Although their energies are shifted, the nature of excitations of those peaks remains the same as that obtained at *B_z_* = 0.25*B*_0_, as shown in Figures 11 and 12.

In the perpendicular case (*C_s_* symmetry), the evolution of the valence orbitals is entirely different from the parallel one (see Figure 10). The HOMO-1 and HOMO (4*a*^′^ and 1*a*^″^ orbitals) are well separated at zero field; however, they cross and reorder at a field strength of about *B_x_* = 0.24*B*_0_. The same trend is also observed for the LUMO+2 and LUMO+3 (7*a*^′^ and 2*a*^″^ orbitals). Regardless of the rather complicated picture of these orbitals, we shall see again the utility of the MO pair decomposition analysis to elucidate the nature of excitations in the computed absorption spectra of H_2_O subject to a magnetic field applied perpendicular to the molecular plane.

**Figure 13:**
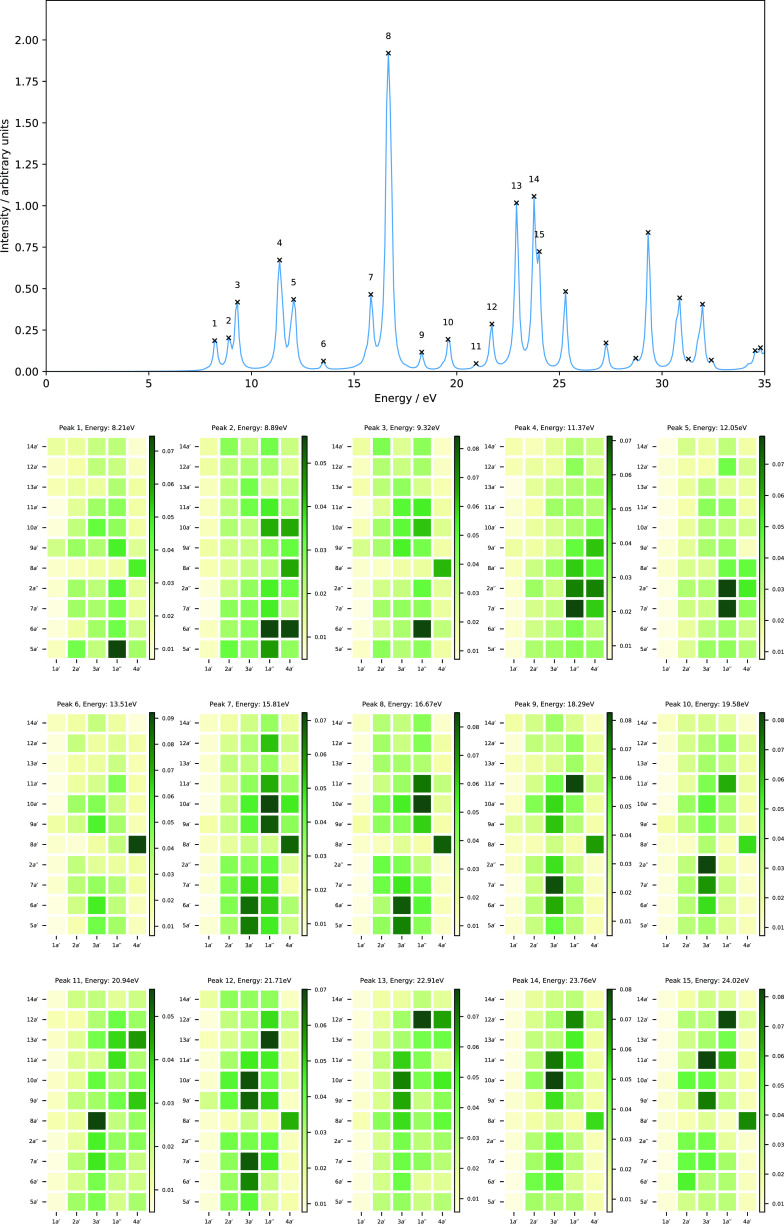
Electronic absorption spectrum and MO pair decomposition analysis for the H_2_O molecule in a magnetic field parallel to the molecular plane, *B_x_* = 0.25*B*_0_, computed using the cTPSSrsh functional and 6-311++G** basis set. The H_2_O molecule is placed in the *yz* plane (molecular plane) with the *z* axis as the *C*_2_ symmetry axis.

**Figure 14:**
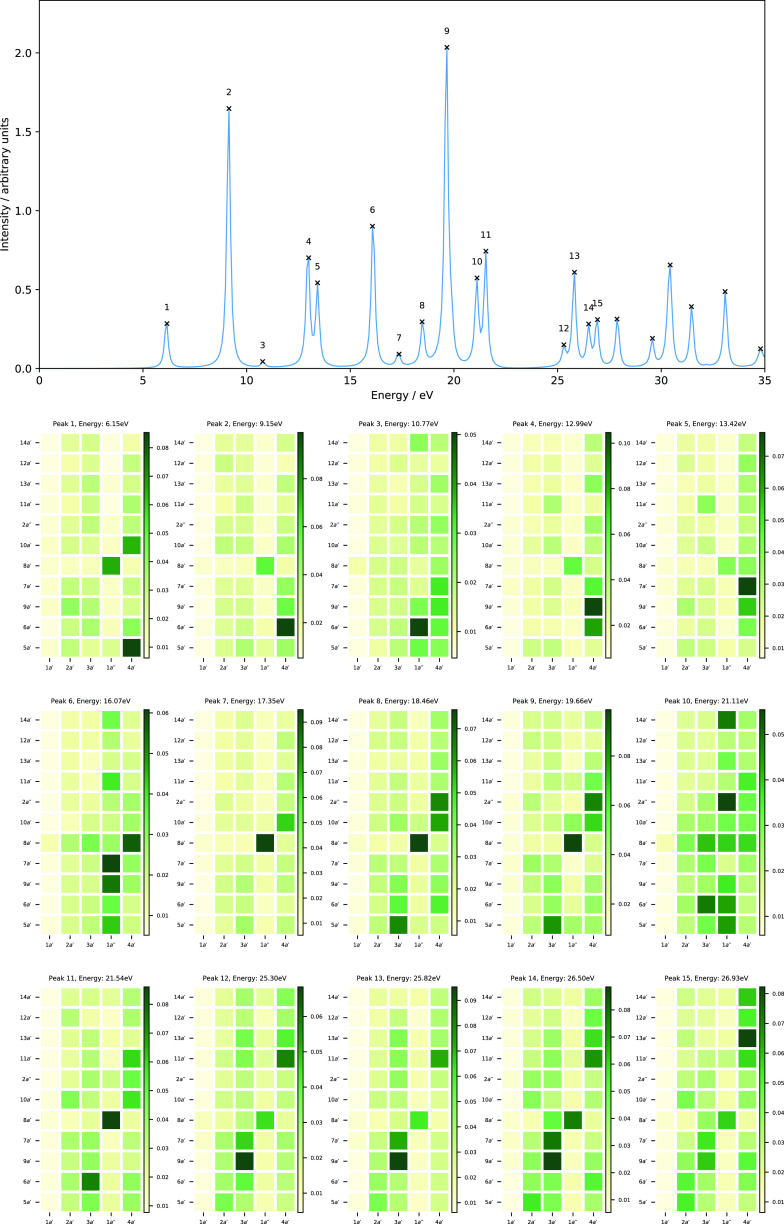
Electronic absorption spectrum and MO pair decomposition analysis for the H_2_O molecule in a magnetic field parallel to the molecular plane, *B_x_* = 0.50*B*_0_, computed using the cTPSSrsh functional and 6-311++G** basis set. The H_2_O molecule is placed in the *yz* plane (molecular plane) with the *z* axis as the *C*_2_ symmetry axis.

Figures 13 and 14 show the electronic absorption spectra and MO pair decomposition analysis for the H_2_O molecule in a magnetic field perpendicular to the molecular plane with *B_x_* = 0.25*B*_0_ and *B_x_* = 0.50*B*_0_, respectively. The corresponding data for the excitation energies and the most dominant transitions are collected in Table 8. At *B_x_* = 0.25*B*_0_, the first valence peak is dominated by a transition with the 1*a*^″^→5*a*^′^ character, which corresponds to peak 1 at zero field and is blue-shifted from the zero-field value by 0.77 eV. The second and third peaks lie close in energy and consist of a dominant transition with the 1*a*^″^→6*a*^′^ character, which would be a forbidden transition at zero field. Peak 2, in addition, also consists of transitions with 4*a*^′^→6*a*^′^ and 1*a*^″^→5*a*^′^ characters. Peaks 4 and 5 show a mixed character with dominant transitions of 1*a*^″^→7*a*^′^ and 1*a*^″^→2*a*^″^ character and a minor contribution from 4*a*^′^→2*a*^″^ for peak 4.

At *B_x_* = 0.50*B*_0_, the mixed character of the first five peaks is less pronounced than those obtained at *B_x_* = 0.25*B*_0_, as shown in Figures 13 and 14. The first and second peaks are dominated by a transition with the 4*a*^′^→5*a*^′^ and 4*a*^′^→6*a*^′^ character, respectively. Peak 1 corresponds to peak 2 at zero field, whilst peak 2 corresponds to peak 3 at zero field. Their energies are red-shifted from the zero-field value by 3.42 and 2.22 eV, respectively. Peak 3 consists of transition with the 1*a*^″^→6*a*^′^ character, corresponding to a forbidden transition at zero field. Peak 4 is dominated by a transition with the 4*a*^′^→9*a*^′^ character and a minor contribution from the 4*a*^′^→6*a*^′^ transition, whilst peak 5 is dominated by the 4*a*^′^→7*a*^′^ transition.

This example shows how the presence of a magnetic field affects the evolution of the MOs and excited-state energies of the H_2_O molecule. Similar to the observations for N_2_, the spectra are significantly altered by application of a magnetic field. Overall, however, the spectra for H_2_O are simpler to interpret since the molecule has a lower symmetry initially and, at zero field, the spectrum is less dense. As a result, the characters of the peaks can be more clearly assigned to specific MO pairs and these can be tracked as a function of the applied field (taking into account the further symmetry lowering by the field).

## 4 Conclusions

In this work, we have presented an implementation of RT-TDHF/RT-TD(C)DFT approaches for molecules in strong magnetic fields. In particular, we have implemented a wide range of propagators for real-time methods and combined these with recent developments such as the MO pair decomposition analysis of Repisky and co-workers.^32^

In combination with our current density functional theory implementation in the QUEST program,^46^ our implementation is capable of full RT-TDCDFT calculations, which explicitly include field-dependent terms in the exchange–correlation functional, in contrast to the implementation of ref (44) where these field dependent exchange–correlation contributions were neglected. This in turn enables the calculation of excitation energies rigorously at the meta-GGA level in line with ref (52) at zero field and seamlessly for magnetic fields of arbitrary strengths. Our previous work has demonstrated that, in such strong magnetic fields, the use of meta-GGA functionals including current-dependent terms can deliver much improved accuracy compared with LDA and GGA type functionals^47,73^ and so provide a good starting point for the determination of excitation energies in strong magnetic fields.

As a first step, we investigated the stability and efficiency of a range of propagators for performing calculations in strong magnetic fields. In line with previous work, we found that the MMUT approach^24^ can provide the best efficiency, though care must be taken to use periodic restarting from other non-leapfrog type propagators, such as the Magnus 2 propagator, to prevent energy drift. We have also implemented the Magnus 2 and Magnus 4 propagators.^62^ Whilst the latter is exceptionally robust, it is not efficient enough for general use due to the large number of Fock/KS matrix constructions required per time step. However, the Magnus 2 approach is more affordable and can be utilized for production runs. We also investigated the EPPC family of propagators.^63^ These approaches allow for the use of larger time steps, with relatively robust performance. In general, we found that the introduction of a magnetic field did not significantly change the relative performance of the propagators and any of these approaches, with appropriate choices of time step and monitoring of energy drift during the simulation, could be applied for production runs.

In the presence of strong magnetic fields, the re-ordering of the molecular orbitals is commonplace as degeneracies are lifted and, at some fields, new accidental degeneracies are created. This has a profound effect on the resulting spectra and so methods for assigning the transitions are essential. We demonstrated the complexities that can arise for the relatively simple N_2_ and H_2_O molecules at the cTPSSrsh/6-311++G** level. Here, the MO pair decomposition analysis^32^ is essential to track the evolution of the spectra as a function of magnetic field, both in terms of the field strength and for different orientations of the applied field. By using the MO pair decomposition analysis,^32^ the features of these can be explained in detail at a level that would be possible from linear response calculations whilst maintaining the advantages of RT-TDCDFT methods for computing the entire absorption spectrum at reasonable computational cost.

An interesting avenue for future work is to explore astrochemical applications, where many simulations are required for the same molecule as a function of field strength and orientation. In this context, RT approaches could provide the entire spectra at low cost, enabling the identification and assignment of interesting transitions that are sufficiently distinct for reliable observation along with their evolution as a function of field. Recent work on enabling the efficient evaluation of molecular gradients in the presence of strong magnetic fields^77^ will also allow for changes in structure to be taken into account. Once the appropriate transitions and spectral ranges are identified, accuracy could be refined by using the more accurate electronic structure methods within the linear-response formalism, such as the recently developed LAO based EOM-CC methods of Stopkowicz and co-workers.^43,74,78^ In this sense, RT-TDCDFT may provide a useful rapid pre-screening tool for studying the spectra of astrochemical species in strong magnetic fields.

In the present work, we have considered a range of functionals based on cTPSS, including hybrid cTPSSh and range-separated hybrid cTPSSrsh variants. Whilst the cTPSS forms have been shown to give accurate results for the ground state of molecules in strong magnetic fields,^47^ their accuracy for excited states in strong fields is yet to be established. Whilst many considerations for exchange–correlation functionals at zero field are expected to carry over to strong magnetic fields, the validation of these functionals should be established in comparison with GW methods^79^ and EOM-CC approaches in this context.^43,74,78^ Finally, the same techniques in the present work have been used to implement chiroptical spectroscopies at the RT-TDCDFT level, which will be presented in future work.

## Supporting Information Available

Details on the efficiency and stability of the propagators (Section 1) and the computed electronic absorption spectra of N_2_ subject to a magnetic field at 45° (*B*_45°_) to the internuclear axis of N_2_ with field strengths ranging from 0.0 to 0.25*B*_0_ (Section 2) (PDF)
