# Supplementary material for: Modeling Ultrafast Electron Dynamics in Strong Magnetic Fields Using Real-Time Time-Dependent Electronic Structure Methods
Source: J Chem Theory Comput. 2021 Mar 16;17(4):2137–65. doi: 10.1021/acs.jctc.0c01269 (PMC8047917; doi:10.1021/acs.jctc.0c01269)
Supplement: Supplementary file 1 — ct0c01269_si_001.pdf [file ct0c01269_si_001.pdf]

Modeling Ultrafast Electron Dynamics in Strong Magnetic Fields Using Real-Time Time-Dependent Electronic Structure Methods

Meilani Wibowo,[†] Tom J. P. Irons,[†] and Andrew M. Teale[*,†,‡]

†*School of Chemistry, University of Nottingham, Nottingham NG7 2RD, United Kingdom*

‡*Hylleraas Centre for Quantum Molecular Sciences, Department of Chemistry, University of Oslo, N-0315 Oslo, Norway*

E-mail: andrew.teale@nottingham.ac.uk

1 Efficiency and stability of time propagators

As supporting information for the discussion on the efficiency and stability of time propagators, we present the average number of Fock / Kohn-Sham matrix constructions per time step for the N_2 molecule in a magnetic field applied parallel to the internuclear axis, $B_{\|} = 0.25B_0$, computed using the EPPC-1 and EPPC-3 algorithms along with various methods and time steps in Table S1. The electronic absorption spectra of N_2 computed using the Magnus 2 and EPPC-1 algorithms along with various levels of theory and time steps are presented in Figures S1 and S2, respectively.

Table S1: Average number of Fock / Kohn-Sham matrix constructions per time step for the N_2 molecule in a magnetic field applied parallel to the internuclear axis, $B_{\parallel} = 0.25B_0$, computed using the EPPC-1 and EPPC-3 algorithms along with various methods and time step.

Δt / a.u.	HF		cTPSS		cTPSSh		cTPSSrsh	
	EPPC-1	EPPC-3	EPPC-1	EPPC-3	EPPC-1	EPPC-3	EPPC-1	EPPC-3
0.05	2.00	2.00	2.00	2.36	2.00	2.00	2.00	2.00
0.10	2.00	2.00	2.00	3.22	2.00	2.42	2.00	2.00
0.20	3.38	2.00	2.00	4.19	3.71	3.84	2.00	2.00
0.25	3.30	2.00	2.00	4.46	2.00	4.32	2.76	2.00
0.50	2.90	2.00	2.41	5.50	2.65	5.63	2.57	2.00
1.00	5.22	2.02	5.79	7.34	5.85	7.26	7.38	2.02

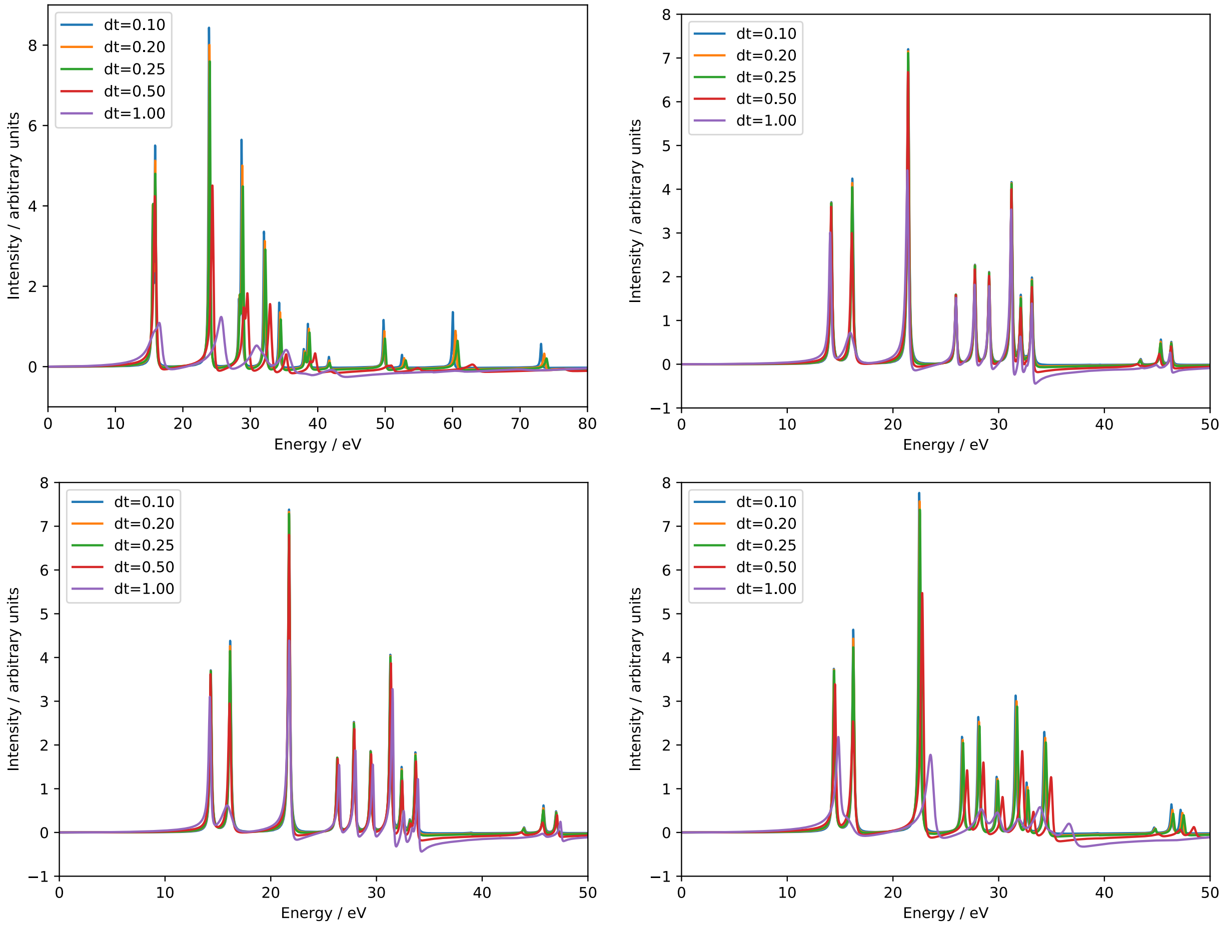

Figure S1: Electronic absorption spectra of N_2 in the absence of a magnetic field, computed using the Magnus 2 algorithm at the HF (top left), cTPSS (top right), cTPSSh (bottom left), and cTPSSrsh (bottom right) levels of theory. All calculations use the 6-31G basis set.

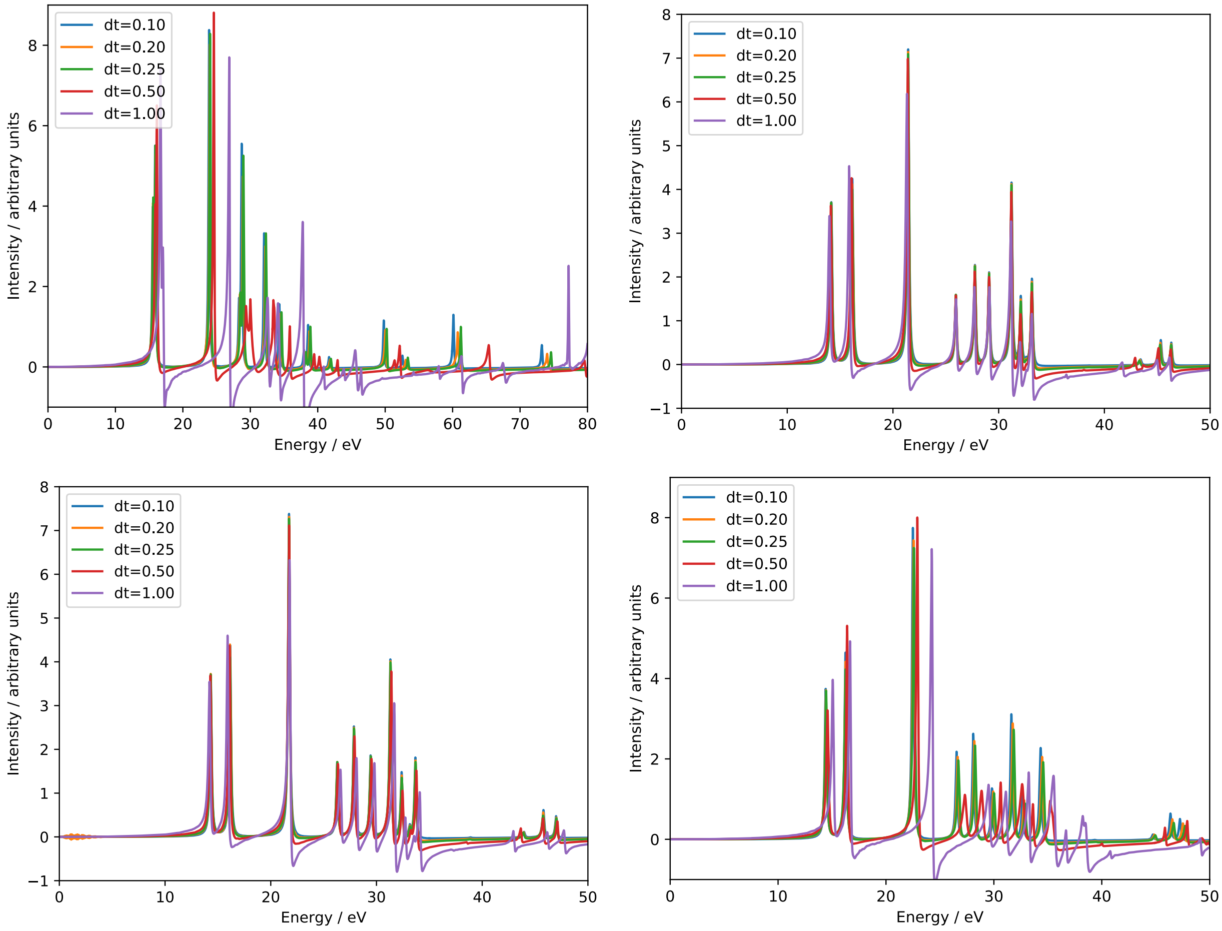

Figure S2: Electronic absorption spectra of N_2 in the absence of a magnetic field, computed using the EPPC-1 algorithm at the HF (top left), cTPSS (top right), cTPSSh (bottom left), and cTPSSrsh (bottom right) levels of theory. All calculations use the 6-31G basis set.

2 Effects of magnetic field on the computed excitation energies

In this section we present the effects of applied magnetic field oriented at 45° to the internuclear axis of the N_2 molecule. The orbital energies as a function of magnetic field oriented at 45° is presented in Figure S3. The first 10 peaks for each field strength $B_{45^\circ} = 0.0$, 0.05, 0.15, and $0.25B_0$ are shown in Table S2. The computed electronic absorption spectra for each field strength $B_{45^\circ} = 0.0$, 0.05, 0.15, and $0.25B_0$ are shown in Figures S4, S5, and S6, respectively.

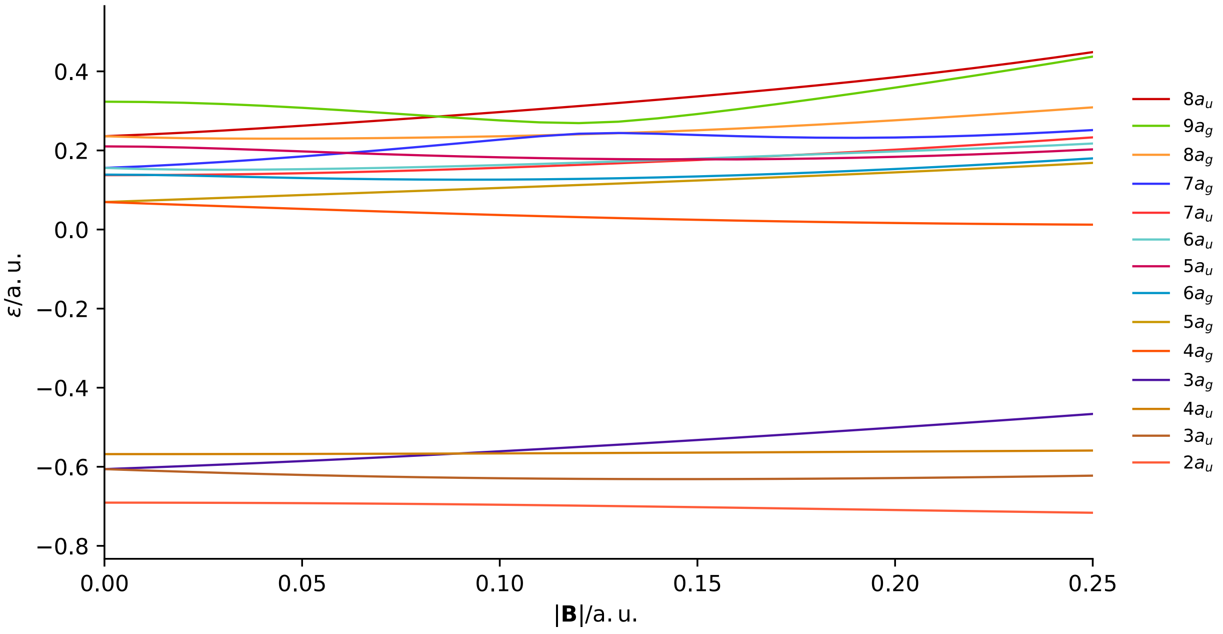

Figure S3: Molecular orbital energies as a function of magnetic fields oriented at 45° to the internuclear axis of the N_2 molecule. All calculations at the cTPSSrsh/6-311++G** level.

Table S2: Excitation energies (ΔE in eV) and dominant orbital characters from the MO pair decomposition analysis for N_2 in a magnetic field oriented at 45° to the internuclear axis. All calculations at the cTPSSrsh/6-311++G** level.

	$B_{45^\circ} = 0.00B_0$		$B_{45^\circ} = 0.05B_0$		$B_{45^\circ} = 0.15B_0$		$B_{45^\circ} = 0.25B_0$	
Peak	Character	ΔE	Character	ΔE	Character	ΔE	Character	ΔE
1	$3a_g \rightarrow 6a_g$	13.68	$2a_u \rightarrow 5a_u$	13.34	$3a_g \rightarrow 4a_g$	9.23	$3a_g \rightarrow 4a_g$	8.98
	$3a_g \rightarrow 5a_u$		$3a_g \rightarrow 6a_g$				$4a_u \rightarrow 5a_g$	
							$4a_u \rightarrow 7a_g$	
2	$3a_g \rightarrow 6a_g$	13.93	$3a_g \rightarrow 5a_u$	13.59	$4a_u \rightarrow 4a_g$	12.31	$4a_u \rightarrow 5a_u$	11.54
	$3a_g \rightarrow 5a_u$		$2a_u \rightarrow 5a_u$		$3a_u \rightarrow 5a_g$		$4a_u \rightarrow 6a_u$	
	$2a_u \rightarrow 5a_u$				$3a_g \rightarrow 5a_g$			
	$3a_g \rightarrow 6a_u$				$3a_g \rightarrow 7a_g$			
	$3a_g \rightarrow 7a_u$							
3	$2a_u \rightarrow 5a_u$	14.19	$3a_g \rightarrow 5a_u$	13.93	$3a_u \rightarrow 5a_g$	12.65	$4a_u \rightarrow 7a_g$	12.65
	$3a_g \rightarrow 5a_u$		$4a_u \rightarrow 6a_g$					
	$3a_g \rightarrow 6a_g$							
	$2a_u \rightarrow 4a_g$							
4	$3a_g \rightarrow 5a_u$	14.62	$4a_u \rightarrow 6a_g$	14.62	$4a_u \rightarrow 7a_u$	13.08	$4a_u \rightarrow 6a_u$	13.25
	$2a_u \rightarrow 5a_u$		$4a_u \rightarrow 6a_u$		$4a_u \rightarrow 6a_u$			
					$3a_g \rightarrow 6a_g$			
5	$3a_u \rightarrow 7a_g$	16.67	$4a_u \rightarrow 6a_g$	15.04	$4a_u \rightarrow 5a_u$	13.42	$4a_u \rightarrow 7a_u$	14.10
	$4a_u \rightarrow 7a_g$		$4a_u \rightarrow 6a_u$					
	$2a_u \rightarrow 5a_u$							
	$3a_u \rightarrow 7a_u$							
	$4a_u \rightarrow 6a_u$							
	$2a_u \rightarrow 6a_g$							
	$3a_u \rightarrow 6a_u$							
	$4a_u \rightarrow 7a_u$							
6	$2a_u \rightarrow 6a_g$	17.10	$3a_u \rightarrow 6a_u$	15.81	$3a_g \rightarrow 6a_u$	14.28	$3a_g \rightarrow 7a_g$	14.53
	$2a_u \rightarrow 5a_u$		$3a_u \rightarrow 7a_u$		$3a_g \rightarrow 7a_g$		$3a_g \rightarrow 5a_g$	
7	$2a_u \rightarrow 7a_g$	19.06	$3a_g \rightarrow 9a_g$	16.75	$3a_g \rightarrow 7a_u$	14.70	$3a_g \rightarrow 7a_g$	15.39
	$3a_g \rightarrow 8a_u$		$2a_u \rightarrow 5a_u$		$3a_u \rightarrow 6a_g$		$3a_g \rightarrow 5a_g$	
	$4a_u \rightarrow 9a_g$		$2a_u \rightarrow 7a_u$				$3a_u \rightarrow 7a_g$	
	$4a_u \rightarrow 8a_g$		$3a_u \rightarrow 7a_u$				$3a_u \rightarrow 6a_g$	
	$3a_u \rightarrow 8a_g$							
	$3a_u \rightarrow 9a_g$							
8	$2a_u \rightarrow 8a_g$	20.00	$2a_u \rightarrow 6a_u$	17.35	$3a_u \rightarrow 7a_u$	15.90	$3a_g \rightarrow 6a_u$	15.64
	$2a_u \rightarrow 9a_g$		$2a_u \rightarrow 6a_g$		$4a_u \rightarrow 7a_g$			
					$3a_u \rightarrow 6a_g$			
					$2a_u \rightarrow 5a_g$			
9	$3a_g \rightarrow 9a_u$	25.73	$2a_u \rightarrow 6a_u$	17.69	$4a_u \rightarrow 9a_g$	16.93	$3a_u \rightarrow 5a_u$	16.67
	$2a_u \rightarrow 13a_g$		$2a_u \rightarrow 6a_g$		$3a_u \rightarrow 7a_g$		$3a_g \rightarrow 6a_g$	
	$2a_u \rightarrow 6a_g$				$3a_u \rightarrow 7a_u$			
					$2a_u \rightarrow 6a_g$			
					$3a_u \rightarrow 5a_u$			
10	$3a_g \rightarrow 11a_u$	29.83	$4a_u \rightarrow 8a_u$	18.81	$2a_u \rightarrow 6a_g$	17.52	$3a_g \rightarrow 8a_g$	17.18
	$3a_g \rightarrow 10a_u$							
	$4a_u \rightarrow 10a_u$							
	$3a_u \rightarrow 11a_u$							
	$3a_u \rightarrow 10a_u$							
	$4a_u \rightarrow 11a_u$							
	$3a_g \rightarrow 10a_g$							

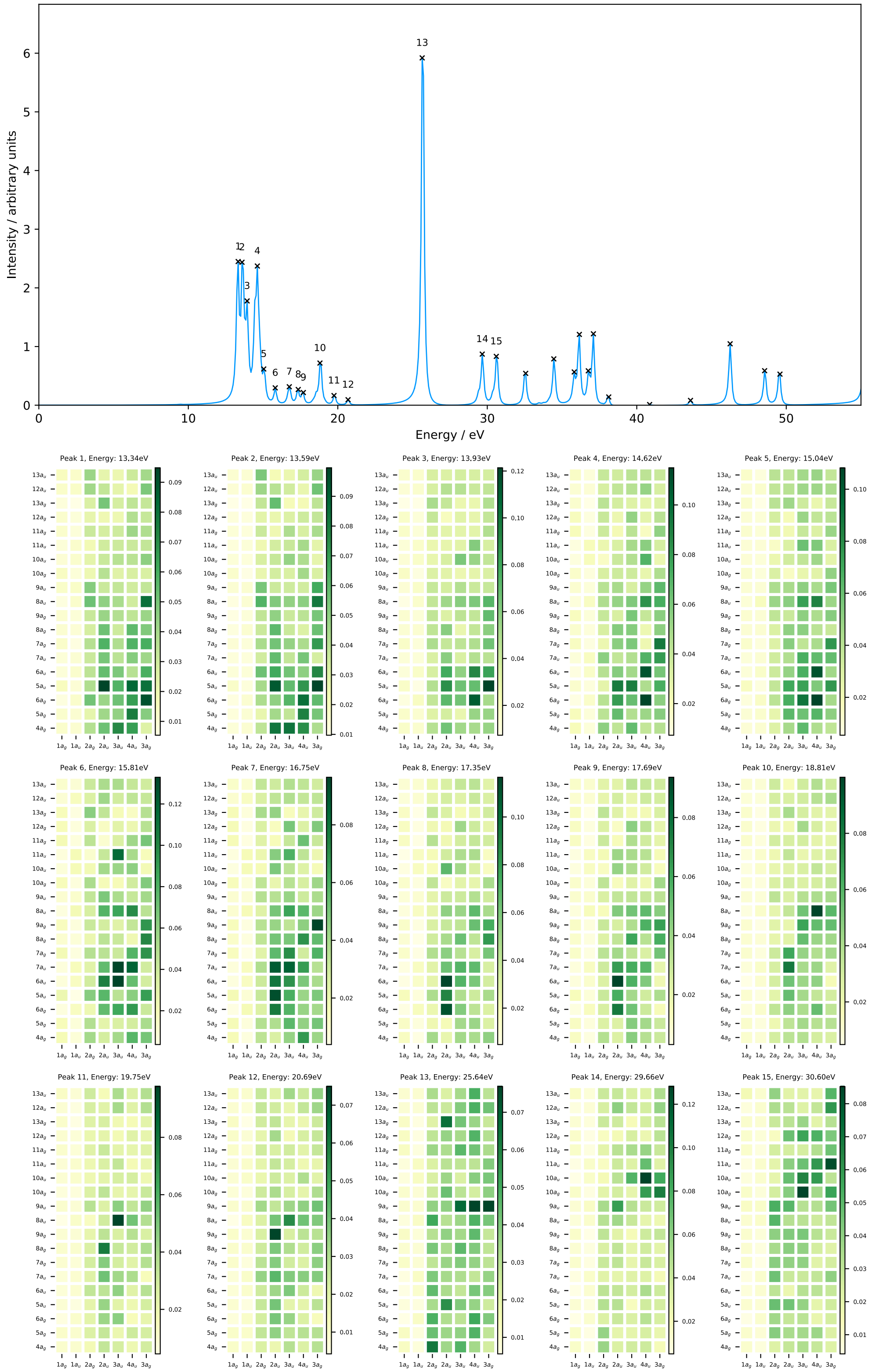

Figure S4: Electronic absorption spectrum and MO pair decomposition analysis for the N_2 molecule in magnetic field oriented at 45° to the internuclear axis, $B_{45^\circ} = 0.05B_0$, computed using the cTPSSrsh functional and 6-311++G** basis set.

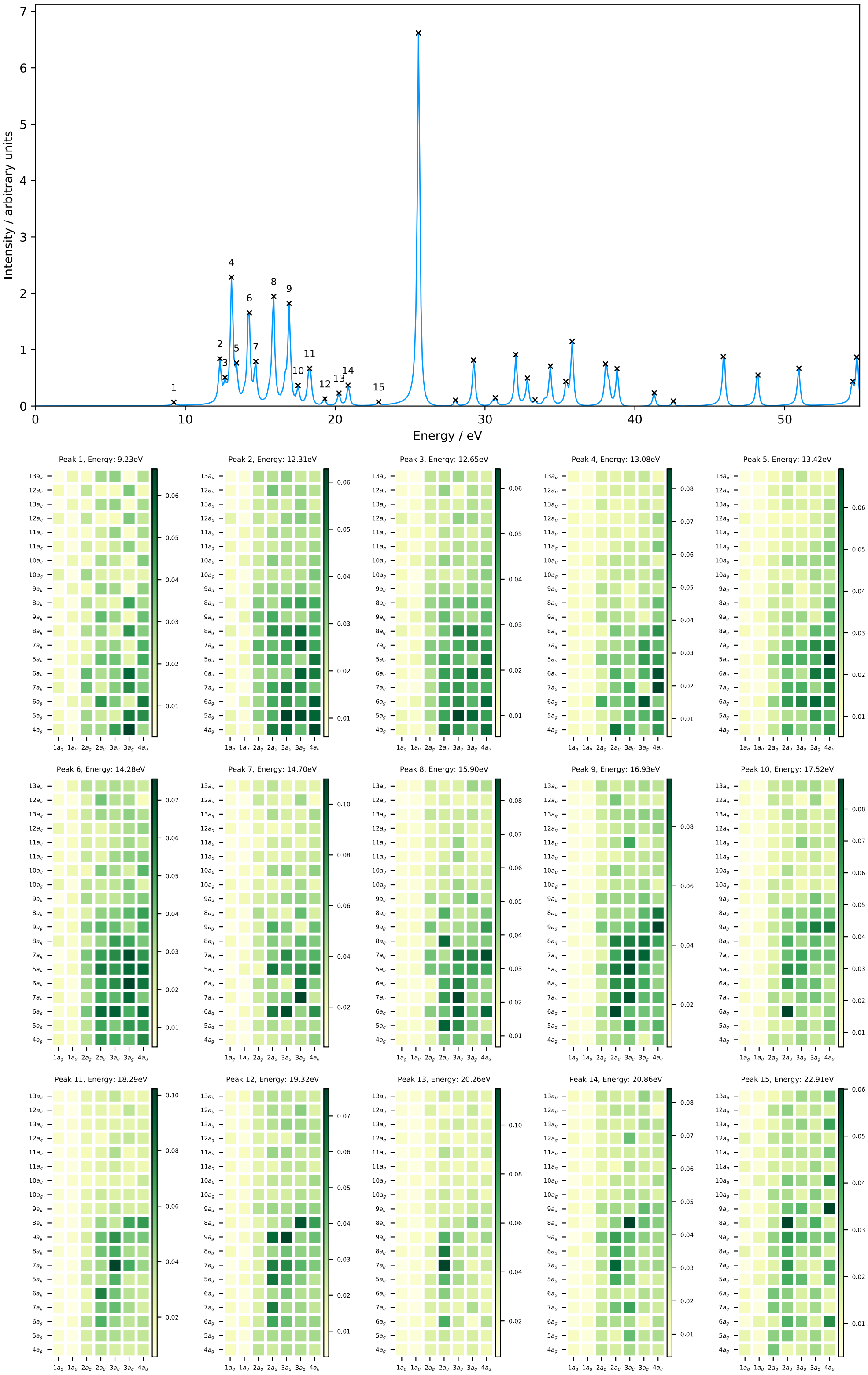

Figure S5: Electronic absorption spectrum and MO pair decomposition analysis for the N_2 molecule in magnetic field oriented at 45° to the internuclear axis, $B_{45^\circ} = 0.15B_0$, computed using the cTPSSrsh functional and 6-311++G** basis set.

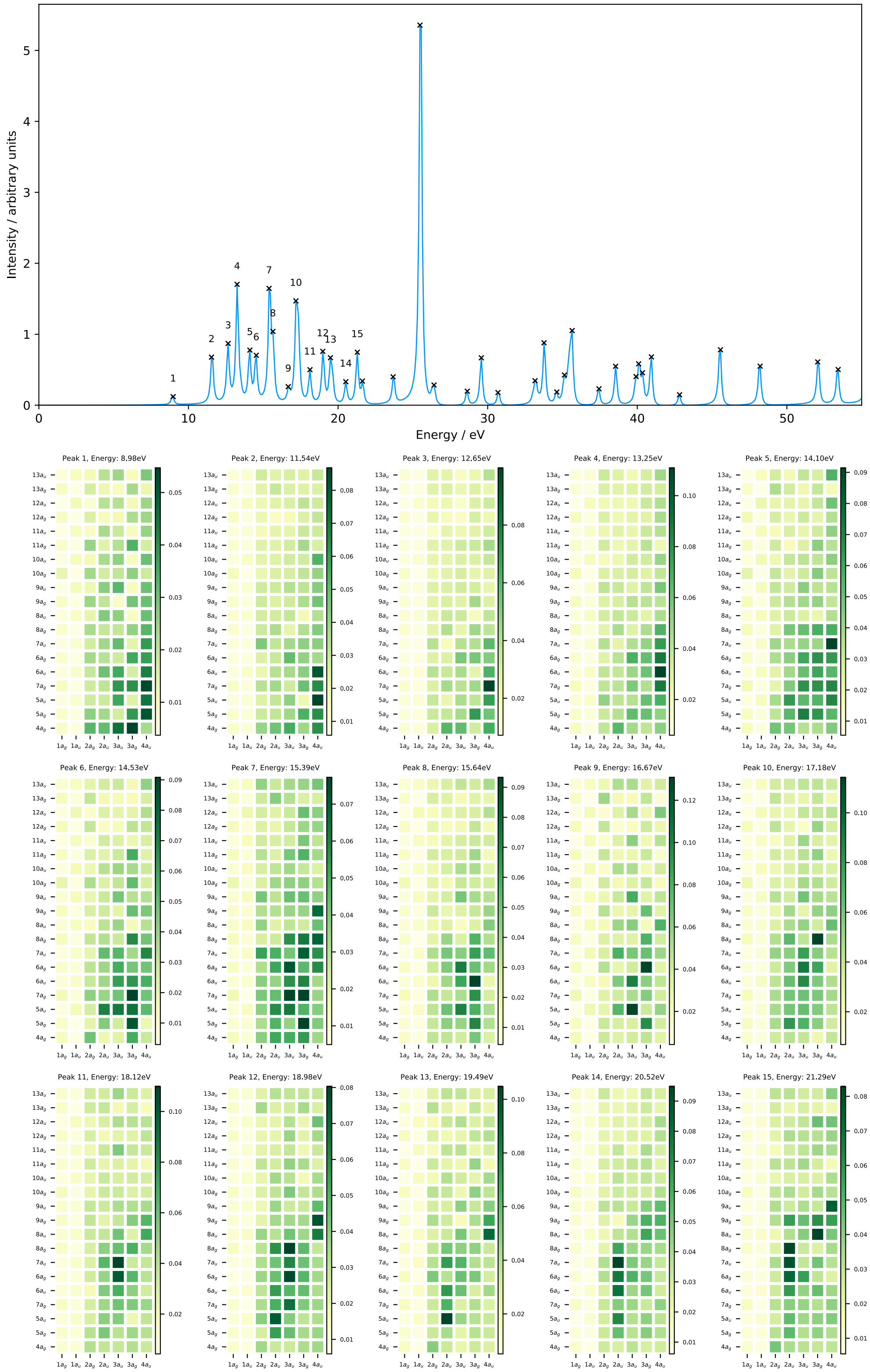

Figure S6: Electronic absorption spectrum and MO pair decomposition analysis for the N_2 molecule in magnetic field oriented at 45° to the internuclear axis, $B_{45^\circ} = 0.25B_0$, computed using the cTPSSrsh functional and 6-311++G** basis set.
